# ^33^S NMR: Recent Advances and Applications

**DOI:** 10.3390/molecules29143301

**Published:** 2024-07-12

**Authors:** Ioannis P. Gerothanassis, Leonid B. Kridvin

**Affiliations:** 1 Section of Organic Chemistry and Biochemistry, Department of Chemistry, University of Ioannina, GR-45110 Ioannina, Greece; 2 A.E. Favorsky Irkutsk Institute of Chemistry, Siberian Branch of the Russian Academy of Sciences, Favorsky St. 1, 664033 Irkutsk, Russia

**Keywords:** ^33^S NMR, chemical shifts, quadrupolar relaxation, solid state NMR, DFT calculations

## Abstract

The purpose of this review is to present advances and applications of ^33^S NMR, which is an underutilized NMR spectroscopy. Experimental NMR aspects in solution, chemical shift tendencies, and quadrupolar relaxation parameters will be briefly summarized. Emphasis will be given to advances and applications in the emerging fields of solid-state and DFT computations of ^33^S NMR parameters. The majority of the examples were taken from the last twenty years and were selected on the basis of their importance to provide structural, electronic, and dynamic information that is difficult to obtain by other techniques.

## 1. Introduction

Sulfur is a widely distributed element in organic, inorganic, polymeric, industrial, and biological systems. It is the 16th most abundant element in the crust of the earth by mass and, thus, an essential component of minerals. Sulfur is transported, with a variety of oxidation states from −2 to +6, by fluids and melts during a wide range of geological processes. Among the four sulfur stable isotopes (^32^S, 94.8%; ^34^S, 4.37%; ^33^S, 0.76%; and ^36^S, 0.02%), only ^33^S possesses a nuclear spin and, thus, is NMR active. Compared to ^1^H, ^13^C, ^15^N, ^31^P, and ^19^F NMR, however, ^33^S has received little attention since it suffers from low natural abundance (0.76%, Table 1), a low gyromagnetic ratio (2.055685 × 10^−7^ rad T^−1^ s^−1^), and extremely low absolute sensitivity relative to that of protons (1.72 × 10^−5^) [1,2,3,4,5]. Furthermore, ^33^S is quadrupolar in nature, with a spin quantum number of I = 3/2 and a relatively large nuclear quadrupole moment of −0.0678 × 10^−28^ m^2^. As a result, the interaction between the nuclear quadrupole moment and the electric field gradient at the nucleus provides very effective relaxation mechanisms and, thus, broad linewidths in solution and significant anisotropic broadening of powder line shapes in the solid state.

Recent developments in instrumentation, new pulse sequences and methodologies, especially in the solid state, the large chemical shift range, and the availability of elemental ^33^S (99.8 atom%) as the starting material for ^33^S enrichment have alleviated some of the experimental difficulties. In addition, due to the widespread application of DFT calculations of NMR parameters, an increased use of ^33^S NMR can be foreseen. General features and available experimental data for ^33^S NMR spectroscopy are discussed in a number of earlier comprehensive reviews and compilations. As an example, Barbarella [2] summarized a wide range of applications up to 1992, while Musio [3] published a very comprehensive review that covers all aspects of ^33^S NMR, including experimental conditions, the theoretical background of various NMR parameters, and a wide range of applications up to 2008. 

In the present review, we have attempted to treat both experimental aspects and the theoretical background of ^33^S parameters and a wide range of applications, with particular emphasis on the solid state and computational DFT methods. Most examples are selected from publications in the last 20 years, yet some older but classic ^33^S NMR papers will also be discussed. An attempt will also be made to define areas where ^33^S NMR could provide atomic structural information that is difficult to obtain using other techniques. 

## 2. Revision of Nuclear Properties

**Figure 1:**
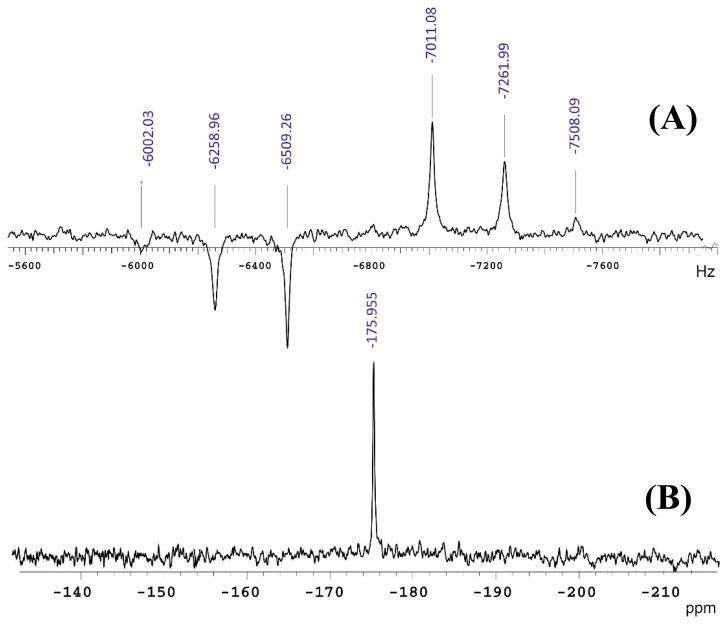
The ^33^S NMR spectra of gaseous SF_6_ at 21.8 atm: (**A**) coupled to six fluorine nuclei, and (**B**) under fluorine decoupling [6].

**Figure 2:**
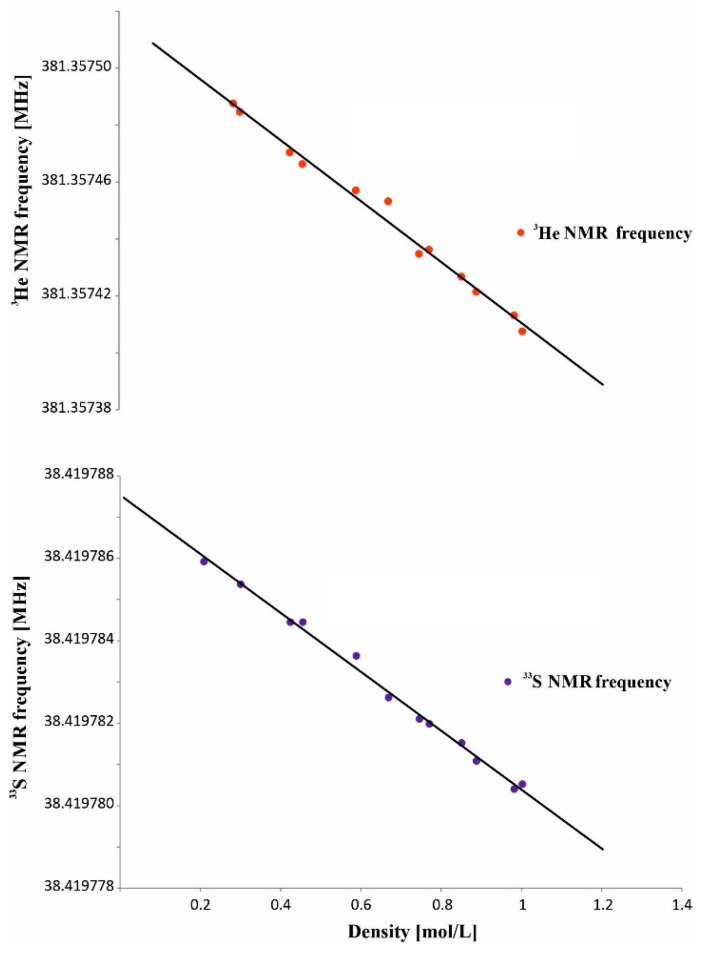
^3^He and ^33^S NMR frequencies of the gaseous mixture ^3^He, SF_6_ as a function of density [6].

Makulski [6] reinvestigated several nuclear dipole moments and magnetic shielding constants, including those of ^33^S, using ^3^He atoms in mixtures with gas molecules of nuclei under investigation. The SF_6_ gas, in the pressure range of 5–25 atm, resulted in very narrow ^33^S signals (Δν_1/2_ ~ 1 Hz), due to the zero electric field gradient on the sulfur nuclei, with a well-defined ^1^*J*(^33^S, ^19^F) = 250.91 Hz (Figure 1). The ^3^He and ^33^S NMR frequencies showed linear density dependencies (Figure 2), which, upon extrapolation to the zero-pressure limit, resulted in a ^33^S nuclear magnetic moment in terms of μ(^3^He). Since the experimental μ(^3^He) is known to have high accuracy, the resulting μ(^33^S) = 0.6432555(10)μ_Ν_ is more accurate by an order of magnitude than those previously reported [7,8] (Table 1). 

## 3. ^33^S Enrichment

**Scheme 1:**
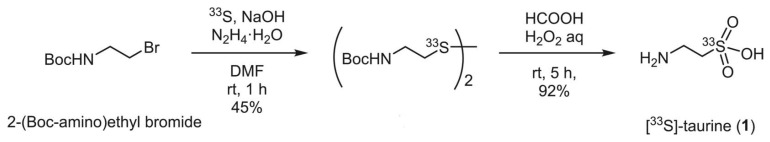
Synthesis of [^33^S]-taurine (**1**). Reprinted with permission from [9]. Copyright 2023, John Wiley & Sons Ltd., Chichester, West Sussex, UK.

The stringent requirements in ^33^S NMR studies, at natural abundance, are the extensive signal averaging and the high concentrations needed for a successful spectrum. In most cases, especially for biologically relevant low concentrations, the use of [^33^S]-labeled compounds is necessary. The synthesis of labeled compounds usually involves typical inorganic and organic reactions using, in most cases, elemental ^33^S (99.8 atom%) as the starting material. A typical example is the synthesis of [^33^S]-taurine, which was achieved according to Scheme 1: disulfide ions, ^33^$S_{2}^{2-}$, which were prepared by the reaction of elemental ^33^S (99.8 atom%) with NaOH in the presence of hydrazine, reacted with 2-(Boc-amino) ethyl bromide to produce [^33^S_2_]-dissulfide. Treatment with performing acid produced [^33^S]-taurine [9]. [^33^S_2_]-L-cystine was also prepared with a similar synthetic root [10]. Literature procedures were also used for the synthesis of [^33^S]-diphenyl disulfide and its precursor, thiophenol [11].

**Figure 3:**
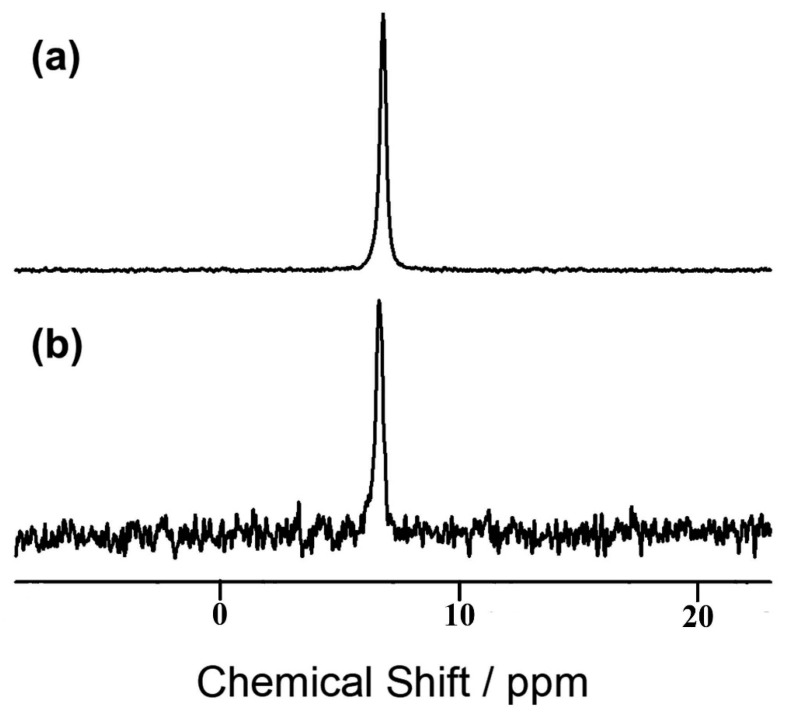
^33^S NMR spectra., at 14.1 T, of: (**a**) 100 mM [^33^S]-taurine in D_2_O, acquisition parameters: spectral width = 1000 ppm, acquisition time = 0.566 s, pulse delay = 1 s, number of scans = 8; (**b**) 0.1 mM [^33^S]-taurine in D_2_O, acquisition parameters: spectral width = 250 ppm, acquisition time = 0.284 s, pulse delay = 0.65 s, number of scans = 40,000. The FID data were multiplied by a 2 Hz exponential line broadening factor prior to Fourier transformation. Adopted with permission from [9]. Copyright 2023, John Wiley & Sons Ltd. The chemical shift range has been corrected relative to saturated (NH_4_)_2_SO_4_ in D_2_O (0 ppm), according to IUPAC recommendation [5].

The ^33^S NMR spectrum of 0.1 mM [^33^S]-taurine in D_2_O (δ = −6.7 ppm, Δν_1/2_ = 11.5 Hz), at 14.1 T (see Figure 3), shows a reasonable signal with S/N = 9, using 4 × 10^4^ scans and a total experimental time of ~10.4 h. Taking into consideration that T_1_ ≈ 51 ms, the pulse repetition time, Τ_p_, could be as short as Τ_p_ = 4T_1_ ≈ 250 ms; thus, the total experimental time can be reduced by a factor of ~3.7. In humans, the concentrations of taurine are in the range of 10 to 100 μM in plasma and 10 to 70 μM in skeletal muscles and the heart; ^33^S-labeled taurine, therefore, can be detected in metabolic and pharmacokinetic studies. 

## 4. Experimental NMR Aspects of Liquid Samples 

### 4.1. Ultra-High Magnetic Fields and High Sensitivity Detection Schemes 

The use of ultra-high magnetic fields has significantly increased the sensitivity of ^33^S NMR, in addition to increasing signal dispersion in the case of more complicated systems. The use of ultra-high field instrumentation is also most advantageous in the case of: (a) large molecular weight systems in solution, outside the extreme narrowing condition, since the m = ½ → m = −1/2 component will dominate the spectrum and it is significantly narrower than the other component (see below on quadrupolar relaxation); and (b) in the solid state since the second-order quadrupolar linewidth of the m = ½ → m = −1/2 decreases linearly with the magnetic field B_o_ (see Section on Solid State).

**Figure 4:**
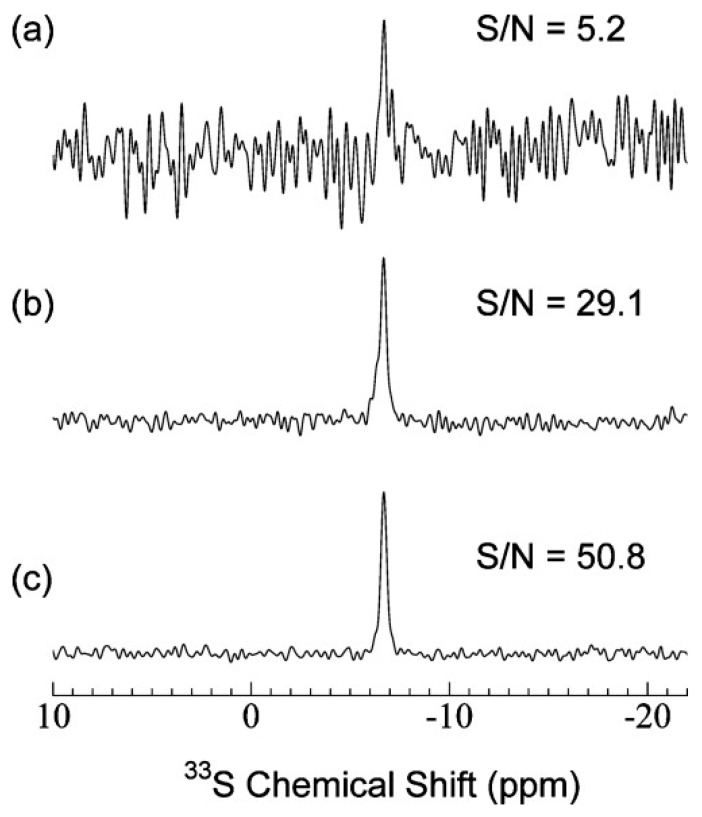
Comparison of the ^33^S NMR sensitivity measured for a 20 mM taurine in D_2_O solution. Acquisition parameters were: spectral width 130 ppm; acquisition time 0.1 s; preacquisition delay⁠ 6–10 μs; relaxation delay 0.05 s; number of scans 40,000 (the corresponding experimental time was 2 h). The NMR spectra were obtained: (**a**) using a conventional 5 mm broadband probe, T = 300 K; (**b**) with the 10 mm cryogenic probe with a room temperature rf switch and a preamplifier, T = 300 K; (**c**) using the 10 mm cryogenic probe with a cold rf switch and a cold preamplifier operated at 60 K. The sample temperature was 292 K. Reprinted with permission from [13]. Copyright 2010, The American Institute of Physics, Melville, NY, USA.

A 10 mm ^33^S cryogenic probe with an inner RF coil in the temperature range of 9–12 K was described as having a 3.5-fold increase in sensitivity relative to that of a conventional 5 mm broadband probe [12]. A further improvement in sensitivity was achieved with a preamplifier and switch that were cooled to 60 K by a cold helium gas [13]. The quality factor Q of the rf coil was increased to 271 compared to 54 at room temperature. Figure 4 shows a comparison of the ^33^S NMR sensitivity using: (a) the conventional 5 mm broadband probe; (b) the 10 mm cryoprobe at room temperature; and (c) the 10 mm cryoprobe with a cold switch and preamplifier. The achievable sensitivity enhancement of ~9.8 in (c) provides a technique to investigate biologically important samples at low mM concentrations for ^33^S nuclei in highly symmetric environments.

### 4.2. Optimization of Experimental Parameters—Effects of Low-Viscosity Solvents and Temperature

In the case of a non-symmetric electronic environment around the ^33^S nucleus, the extremely short quadrupolar relaxation times result in extensive line broadening and, thus, a reduction in the S/N ratio. Sensitivity, however, should be evaluated relative to the achievable S/N ratio in a given period of time. For the broad ^33^S resonance, T_1_ = T_2_, thus significantly short pulse repetition times of ~4T_2_ can be used. This results in an S/N ratio per unit time that is practically independent of T_2_ and, thus, resonance linewidth (see below on the effect of acoustic ringing on the achievable S/N ratio).

Increasing the temperature and/or dilution of high-viscosity liquids with low viscosity solvents can reduce the linewidth of ^33^S. For moderate dilution, an optimum balance in resolution vs. S/N ratio can be achieved [14]. Further reductions in linewidths can be achieved with the use of supercritical solvents, which combine fluid densities with gas-like viscosities. Unfortunately, despite some original optimism, the resolution advantages were significantly inferior to those expected on the basis of the viscosities of supercritical solvents [2]. Thus, no further applications have so far been reported.

### 4.3. Acoustic Ringing 

Observation of broad resonances of low natural abundance, low frequency, and sensitivity in quadrupolar nuclei is very problematic due to rolling baseline artifacts. They were attributed to ultrasonic waves from the effect of the rf. pulses and are often referred to as acoustic ringing [14,15]. The most commonly used method to alleviate the problem is the use of a preacquisition delay, Δt, which inevitably results in a significant reduction in the S/N ratio by a factor of exp(−Δt/T_2_). Thus, resonances with linewidths greater than 4–5 kHz are virtually lost. The most efficient methods for recording very broad resonances are the use of multipulse sequences such as the RIDE and extended spin-echo sequences, which can be routinely applied without any hardware or probe modification [14,15]. More recently, an interactive baseline correction algorithm for solid-state NMR spectra was reported [16]. The method, which can also be applied to liquid states, does not make any hypothesis regarding the NMR line shapes and does not modify the recorded FID data points. 

### 4.4. Magnetization Transfer Experiments—2D Inverse Detection

**Figure 5:**
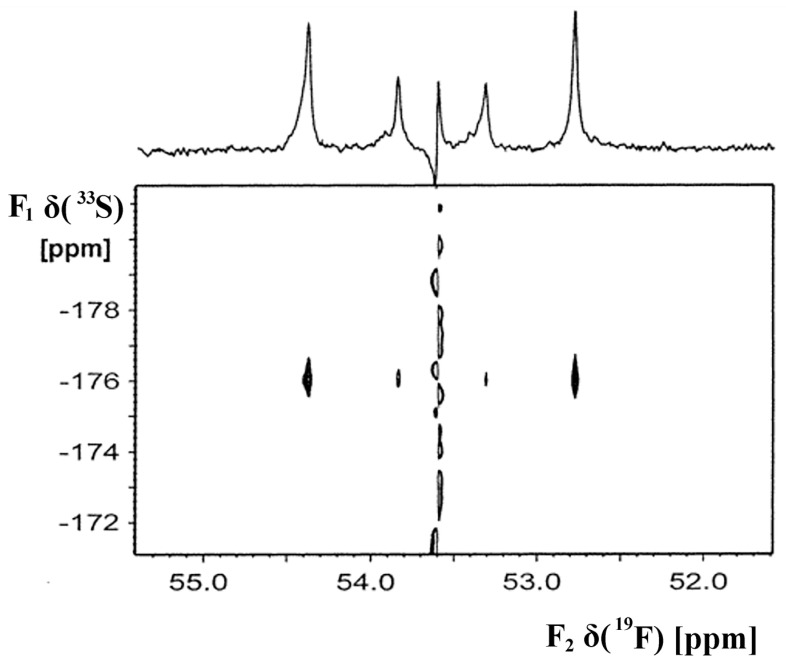
Selected region of a heteronuclear ^33^S/^19^F NMR correlation spectrum of gaseous SF_6_ at high pressure (approximately 20 atm). The 2D spectrum was obtained by the application of the refocused ^33^S-^19^F HMQC technique. Sixteen scans were collected for each TPPI dataset in 180 t_1_ increments. The maximum t_1_ and t_2_ times were 90 and 512 ms, respectively. A relaxation delay of 0.1 s was used. The data matrix containing 180 × 2048 complex points in t_1_ and t_2_ was zero-filled to 512 × 4096 complex points and apodized by a cosine function in both time domains prior to Fourier transformation. Reprinted with permission from [17]. Copyright 2002, The American Institute of Physics, Melville, NY, USA.

Inverse detection schemes in which polarization is transferred from ^1^H to the X nucleus through ^n^*J*(^1^H,X) couplings and then back to ^1^H for detection have proved extremely useful for the I = 1/2 nuclei. In the case of ^33^S, inverse detection was possible only in high-symmetric systems with a zero electric field gradient. Jackowski et al. [17] investigated intermolecular interactions on ^33^S chemical shifts of gaseous SF_6_ and its binary complexes with Xe, CO_2,_ and NH_3_ at 298 K with the use of a refocused ^19^F-^33^S heteronuclear multiple-quantum coherence NMR. This polarization transfer experiment from ^19^F to ^33^S permitted the indirect detection of ^33^S even in low-density samples (Figure 5). This allowed investigation of the density dependence of δ(^33^S) and ^1^*J*(^19^F-^33^S). Similarly, ^19^F-^33^S HMBC experiments were used to investigate SF_6_ dissolved in thermotropic liquid crystals [18].

### 4.5. Referencing Techniques

**Figure 6:**
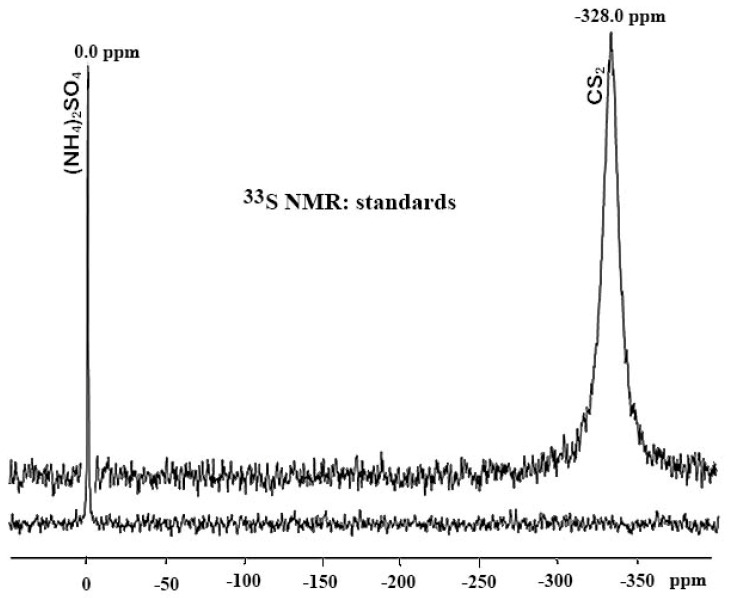
^33^S NMR spectra of saturated (NH_4_)_2_SO_4_ in D_2_O and neat CS_2_. Reproduced with minor editing privilege from http://chem.ch.huji.ac.il/nmr/techniques/1d/row3/s.html under the Creative Commons Attribution 4.0 International Public License. Accessed on 1 June 2024.

For ^33^S NMR, as is common with several heteronuclei, a variety of reference compounds and referencing procedures, both internal and external, have been suggested. There are two adopted scales of ^33^S NMR chemical shifts, namely the one based on neat carbon disulfide, CS_2_, and the other one—on saturated (NH_4_)_2_SO_4_ in D_2_O. The ^33^S NMR signals of both standards are relatively sharp, with the former being noticeably broader because of increased electronic asymmetry around the nucleus, which is illustrated in Figure 6. Despite the IUPAC recommendation of the use of saturated (NH_4_)_2_SO_4_ in D_2_O as a reference compound [5], Cs_2_SO_4_, Na_2_SO_4_, and other sulfates have also been utilized. The ${SO}_{4}^{2^{-}}$ chemical shift was shown to be dependent on concentration, nature of counterion, pH, and temperature. As discussed in detail in [3], the reported chemical shift variations are not significant compared to the accuracy of recording very broad ^33^S resonances. Several authors also still use CS_2_ as a reference. The conversion of the ${SO}_{4}^{2^{-}}$ and the CS_2_ scales was reported to be [3]:

(1) $$\delta ({SO}_{4}^{2^{-}})=\delta ({CS}_{2})-333$$

For diamagnetic solutions, the double tube method was used with the reference compound in the inner tube or capillary, without any susceptibility correction, since it is expected to be much smaller than the accuracy of the measurement of the chemical shift. It should be emphasized, however, that due to the high B_0_ stability of modern NMR instrumentation, the spectrum of the reference compound can be recorded in a separate experiment (when unlocked) with the chemical shift calculated at 0 ppm. For the study of paramagnetic solutions, the use of a cylindrical and a spherical tube has been proposed [14,19]. The ^33^S resonance of the reference compound is recorded in both cells; the bulk magnetic susceptibility correction is the chemical shift difference of the two measurements. 

Recently, Jackowski and Wilczek [20] recommended the use of helium-3 gas as a primary universal reference standard. Gas phase ^3^He NMR measurements provided the resonance frequency of an isolated helium-3 atom, which is independent of temperature, and no rovibration correction is needed. In addition, very accurate shielding constant calculations of σ_ref_(^3^He) are known [21]. The shielding from ^3^H to, e.g., ^33^S can be obtained with the double resonance method using the ^2^D NMR signal of the lock solvent; thus, no reference standard is needed.

## 5. Chemical Shifts 

### Experimental Chemical Shift Ranges 

**Figure 7:**
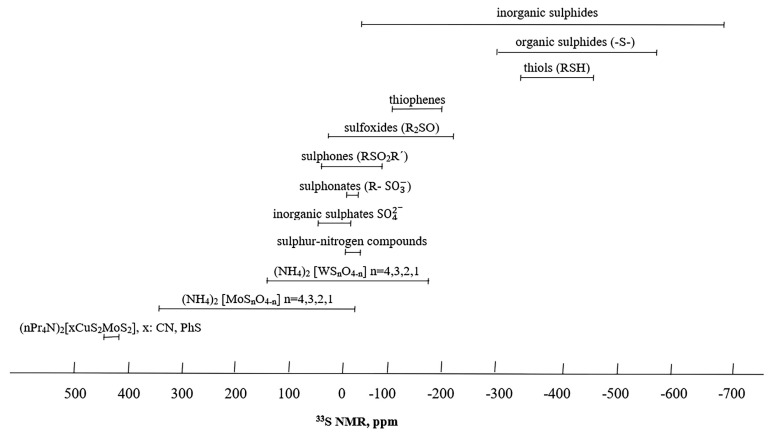
^33^S NMR chemical shift ranges of some of representative sulfur functional groups of diamagnetic organic and inorganic compounds relative to saturated (NH_4_)_2_SO_4_ in D_2_O as reference calibrated at 0 ppm.

The ^33^S NMR scale has a wide chemical shift range of over 1000 ppm, as illustrated in Figure 7, which partly compensates for experimental difficulties in observing the NMR signals. The order of increasing deshielding follows the trend (with several exceptions): (i) singly bonded sulfur; (ii) multiply bonded sulfur; (iii) sulfur belonging to delocalized functional groups; and (iv) sulfur bonded to several oxygen atoms. Inorganic sulphides $\left(S^{2-}\right)$ cover an extremely wide range of −680 ppm (Li_2_S) to −42 ppm (BaS), linear and cyclic organic sulphides (-S-) a region of −573 to −302 ppm, thiols (RSH) −458 to −332 ppm, thiophenes −200 to −113 ppm, sulfoxides (R_2_SO) −213 to 27 ppm, sulfones (RSO_2_R′) −18 to 25 ppm, cyclic sulphones −88 to 42 ppm, sulphonates (R- ${SO}_{3}^{-}$) a very narrow range of −19 to 4 ppm, aromatic sulphonates −19 to 0 ppm, inorganic sulfates (${SO}_{4}^{2-})$ −13 to 67 ppm and sulfur-nitrogen compounds (sulphimides, sulphoximides and sulphonamides) −39 to 2 ppm. There are several miscellaneous organic and inorganic compounds that exhibit highly shielded or deshiled ^33^S resonances, such as OCS (−594 ppm), PhNCS (−570 ppm), (CH_3_)_3_PS (−536 ppm), SO_2_ (374.9 ppm), (NH_4_)_2_ [MoS_n_O_4−n_]: n = 4 (344 ppm), n = 3 (240 ppm), n = 2 (123 ppm), n = 1 (−25 ppm), and (nPr_4_N)_2_[xCuS_2_MoS_2_], x: CN (445 ppm), x = PhS (436 ppm).

As noted above, the ^33^S NMR linewidth is dependent on the magnitude of the electric field gradient at the sulfur nucleus, which in turn depends on the degree of symmetry around sulfur. Thus, while narrow lines are observed for compounds with high molecular symmetry, compounds with lower molecular symmetry, such as sulfoxides and sulfides, give much broader lines. Table 2 and Table 3 contain experimental ^33^S NMR chemical shifts and line widths of several representative sulfides, sulfoxides, and sulfones compiled from the reviews by Musio [3] (compounds **1**–**12**, **28**–**33**), Aliyev [22] (compounds **13**–**16**), and the original paper by Ngassoum et al. [23] (compounds **17**–**27**, **34**–**38**). Most of the original experimental data (which were largely not included in those tables) were published in the early papers by different authors; see most representative reviews [1,2,3] and key original references given therein. 

It appears that δ(^33^S) is sensitive to the electronic effects of substituents, molecular conformation, ring strain, ionization state, temperature, solvent, and, in a few cases, concentration. Characteristic structural trends dealing with the ring strain of cyclic sulfides, sulfoxides, and sulfones were explicitly formulated by Musio [3]. By comparing dimethyl derivatives and the corresponding thiiranes, significant shielding is observed upon cyclization due to the influence of ring strain tension (−145 ppm for thiirane, −205 ppm for thiirane 1-oxide, and −75 ppm for thiirane 1,1-dioxide). Three-membered ring compounds are markedly more shielded than larger rings. On going from three- to four-membered rings, a significant deshielding is observed; from four- to five-membered rings, a further deshielding is observed only in the case of sulphonyl compounds. ^33^S is more shielded in six-membered rings than in five-membered rings by approximately 40–50 ppm, and, thus, it occurs in the same range as the open-chain compounds. This can be attributed to the geometric stability of six-membered rings and the absence of ring-strain interactions.

Several empirical correlations have been reported, which have been analyzed in detail in the comprehensive review of Musio [3]. Thus, only a brief account will be given below. A linear correlation between δ(^33^S) of sulphonates, R${SO}_{3}^{-}$, and δ(^33^C) of carboxylates, RCOO^−^, was obtained in the form

δ(^33^S) = −390.37 + 2.129 δ(^13^C) (2)

which shows that δ(^33^S) is two times more sensitive to substituent effects than that of δ(^13^C). Good linear correlations between δ(^33^S) and Taft polar substituent constant σ* were obtained for symmetric dialkyl sulphones of the form

δ(^33^S) = −155 σ* − 133 (3)

and monosubstituted dimethyl sulfones of the form

δ(^33^S) = 8.78 σ* − 18 (4)

In the last fifteen years, there has been no significant research activity on empirical correlations, presumably due to the fact that theoretical calculations of ^33^S chemical shifts can provide accurate results and, thus, important information on electron distribution and bonding properties (see Section on Computations). 

## 6. Relaxation Properties

### 6.1. Relaxation in the Extreme Narrowing Condition 

In the absence of chemical exchange, the linewidth of quadrupolar nuclei in isotropic systems is given by

(5) $${\Delta \nu }_{1/2}=\frac{1}{\pi {Τ}_{1}}=\frac{1}{\pi {Τ}_{2}}=\frac{3\pi }{10}\frac{2Ι+3}{{Ι}^{2}(2Ι-1)}C_{Q}^{2}\left(1+\frac{\eta_{Q}^{2}}{3}\right)\tau_{c}$$

where T_1_ and T_2_ are the longitudinal and transverse relaxation times, respectively, $C_{Q}$ is the nuclear quadrupolar coupling constant (NQCC) defined as

*C_Q_* = e^2^*q_zz_* Q/h (6)

where Q is the nuclear electric quadrupole moment, *q_zz_* is the largest component of the electric field gradient tensor at the ^33^S nucleus, e is the charge of the electron, $n_{Q}$ is the asymmetric parameter given by

(7) $$n_{Q}=\frac{q_{xx}-q_{yy}}{q_{zz}},\;\left|q_{zz}\right|\geq \left|q_{yy}\right|\geq \left|q_{xx}\right|$$

and $\tau_{c}$ is the correlation time, which for spherical molecules is given by the Stokes-Debye formula.

(8) $$\tau_{c}=\frac{4\pi r^{3}n_{v}}{3K_{B}T}$$

where r is the radius of the molecule, *n*_v_ is the viscosity of the solution, K_B_—the Boltzmann factor, and T—the absolute temperature.

Narrow resonances can be expected for small molecular weight molecules and $C_{Q}$ values at elevated temperatures and low viscosity values. ^33^S NMR signals of a few Hz can only be observed in highly symmetric electronic environments around the sulfur nucleus, such as ${SO}_{4}^{2^{-}}$ anions. The product $C_{Q}^{2}$(1 + $\eta_{Q}^{2}$/3) can be obtained from ^33^S relaxation times when the correlation time can be estimated through Equation (8). More precise values of τ_c_ can be obtained from relaxation time measurements of other nuclei, such as ^13^C and ^2^D, denoted as the dual spin probe method. Thus, the ^33^S NQCCs of the C_6_H_4_${SO}_{3}^{-}$ (0.5 MHz) and 3-${NH}_{3}^{+}$C_6_H_4_${SO}_{3}^{-}$ (1.0 MHz) anions were determined with the combined use of ^33^S linewidths, ^13^C T_1_ relaxation times, and NOE measurements [24]. The ^33^S NQCCs of (CH_3_)_2_SO_2_ (1.8 MHz) and CS_2_ (13.8 MHz) were measured in liquid crystalline solvents [25]. The order parameters were obtained from ^1^H NMR spectra for (CH_3_)_2_SO_2_ and ^13^C chemical shifts for CS_2_. Information on the NQCC of (CH_3_)_2_SO_2_ in chloroform solution was also obtained (1.63 MHz for $n_{Q}$= 0 and 1.41 MHz for $n_{Q}=$ 1) with the combined use of ^33^S and ^2^D T_1_ relaxation time measurements. The values in chloroform solution are lower than those reported for liquid crystalline solvents, presumably due to solvent effects and experimental errors [25]. In the last twenty years, however, the great majority of ^33^S NQCCs were obtained with solid-state and zero-field NMR techniques (see Section 8 on Solid State and Section 9 on Computations). 

### 6.2. Relaxation Outside the Extreme Narrowing Condition 

The decay of the longitudinal and transverse magnetization components outside the extreme narrowing condition, ω_0_$\tau_{c}$ >> 1, is no longer exponential but a weighted sum of I + 1/2 = 2 exponentials. The NMR signal of the m = 1/2 → m = −1/2 component will dominate the spectrum because it is significantly narrower than the other component [26,27]. This phenomenon has been successfully used in ^17^O NMR to investigate ligand binding to proteins [14] or small MW molecules dissolved in highly viscous solvents [28]. Unfortunately, no application of ^33^S NMR has so far been reported in solution, contrary to the case in the solid state (see Section 8 on Solid State). 

## 7. Selected Applications in Solution

### 7.1. Biological Applications 

**Figure 8:**
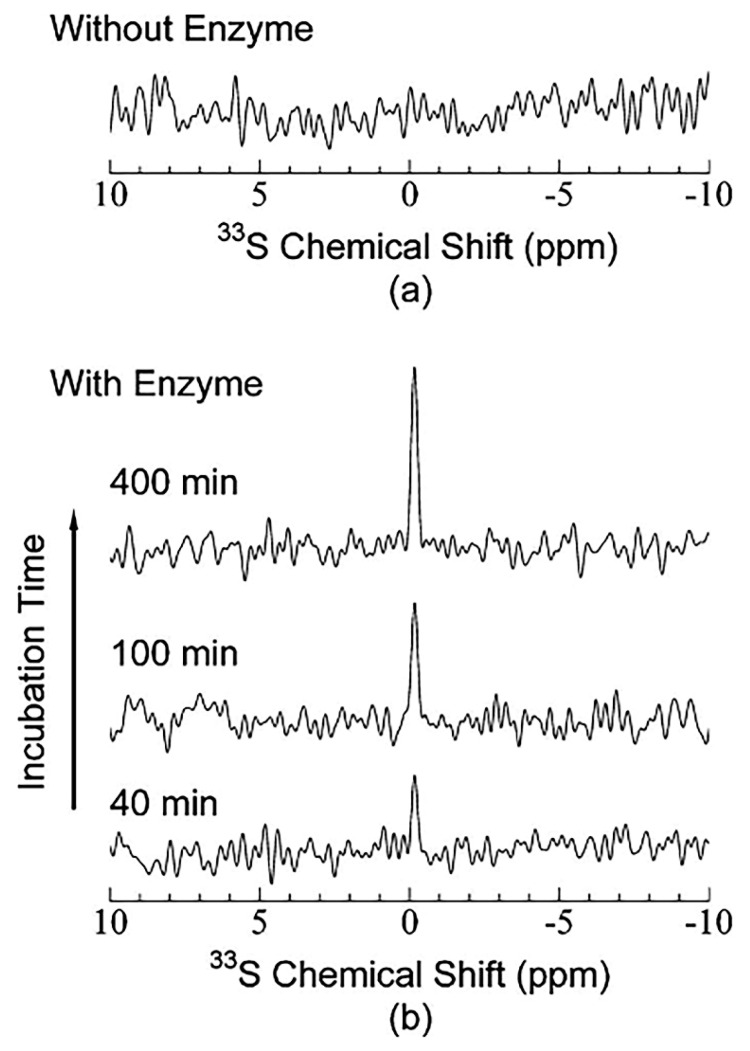
(**a**) ^33^S NMR spectrum of chondroitin sulfate A(ChS-A) in the absence of the enzyme chondroitinase-ACII Arthro. (**b**) ^33^S NMR spectra of chondroitin sulfate A with the enzyme; the parameters listed are the incubation times of the enzyme catalyzing reaction. The NMR spectra were obtained with the use of a ^33^S cryogenic probe, a room-temperature rf switch, and a room-temperature preamplifier. Acquisition parameters: spectral width 130 ppm; acquisition time 0.1 s; preacquisition delay 10 μs; and relaxation delay 0.01 s. Total number of scans: 400,000, and experimental time: 16 h, 10 min. The sample temperature was in the range of 290–292 K. Reprinted with permission from [13]. Copyright 2010, The American Institute of Physics, Melville, NY, USA.

**Figure 9:**
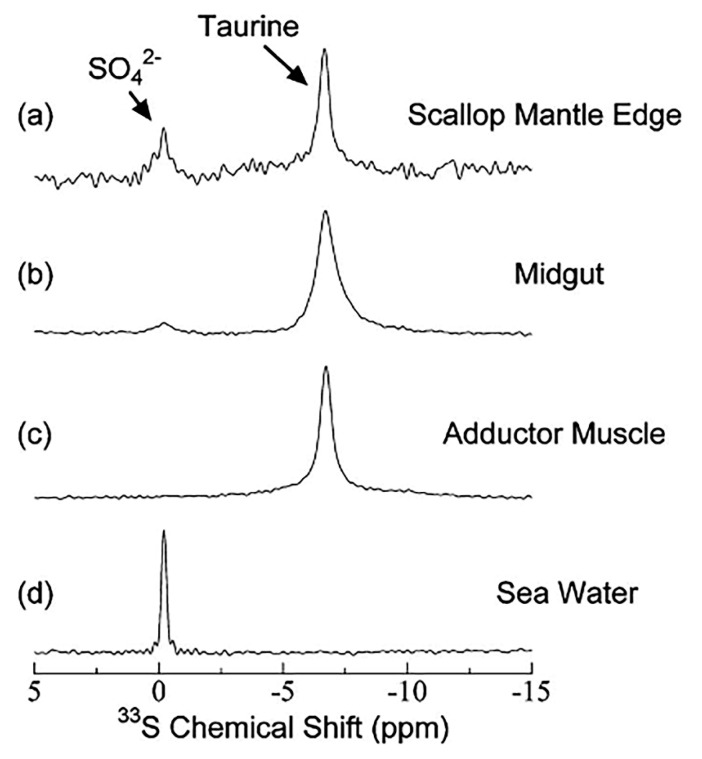
^33^S NMR spectra of (**a**) mantle edge, (**b**) midgut, and (**c**) adductor muscles of a scallop, and (**d**) of sea water. The spectra were obtained with the use of the ^33^S cryogenic probe with a cold rf switch and a cold preamplifier (60 K). The acquisition parameters were the same as in Figure 8. 400,000 scans were recorded, and the experimental time was 16 h 10 min. Sample temperature: 291–292 K. Reprinted with permission from [13]. Copyright 2010, The American Institute of Physics, Melville, NY, USA.

^33^S was used to investigate the effect of the enzyme chondroitinase (ACII), a bacterial lyase that specifically digests chondroitin sulfate A (ChS-A), which is a sulfated glycosaminoglycan of over 100 residue disaccharides, each of which can be sulfated in variable positions and quantities [13]. ChS-A, in the absence of ACII, shows no observable ^33^S NMR signal due to extensive linewidth broadening. With an increase in the incubation time and, thus, enzymatic catalysis, a sharp peak at −0.5 ppm appeared (Figure 8). This was attributed to ${SO}_{4}^{2^{-}}$ species produced due to the enzymatic cleavage of the N-acetylhexosaminide linkage in ChS-A. ^33^S NMR was also utilized to investigate the tissue of the mantle edge, midgut, and adductor muscles of a scallop. ${SO}_{4}^{2^{-}}$ species were clearly identified relative to the taurine resonance (Figure 9). 

### 7.2. ^77^Se as a Substitution for Sulfur—^1^H Detected ^77^Se NMR in Proteins 

^77^Se enrichment of proteins has been used as a tool to expand the biological applications of NMR [29] based on the great sensitivity of ^77^Se to electronic environments and dynamics [4]. Recently, sulfur sites with double-labeled ^13^C and ^77^Se proteins were explored with the use of triple resonance ^1^H-detected ^77^Se NMR [30]. For the case of double-labeled methionine, the coupling constants ^1^*J*(^1^H, ^13^C) = 125–140 Hz and ^1^*J*(^13^C, ^77^Se) = 60–70 Hz were used for coherence transfers as follows:

^13^CH_3_ → ^13^CH_3_ → ^77^Se → ^13^CH_3_ → ^13^CH_3_

**Figure 10:**
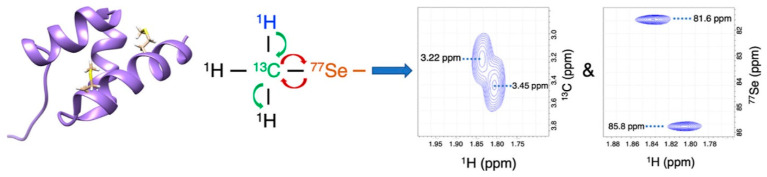
^1^H/^13^C/^77^Se NMR spectra (600 MHz for ^1^H) of ^13^CH_3_^77^Se-methione incorporated protein CUE (MW = 6 kDa) of 300 μM concentration at 20 °C. The ribbon diagram shows the location of the two methionine residues, M467 and M450. Reprinted with permission from [30]. Copyright 2023, The American Chemical Society, Washington, DC, USA.

The great resolution and sensitivity advantages of the method are illustrated in Figure 10 using a concentration of 300 μM of the protein CUE (MW = 6 kDa), in which methionine residues were substituted to ~95% with double-labeled ^13^CH_3_-^77^Se-methionine. Although ^1^H and ^13^C chemical shift differences are very small, the ^77^Se chemical shifts of the methionine residues M467 and M450 show a significant difference of 4.2 ppm. The technique requires specific hardware and decoupling schemes that are not, at present, commercially available. It remains to be seen what the advantages of the method are for proteins with molecular weights larger than 20 kDa since, at ultra-high magnetic fields, the line broadening might be significant due to the very large chemical shift anisotropy of ^77^Se [4]. 

## 8. ^33^S NMR in the Solid State

### 8.1. Basic Considerations

**Figure 11:**
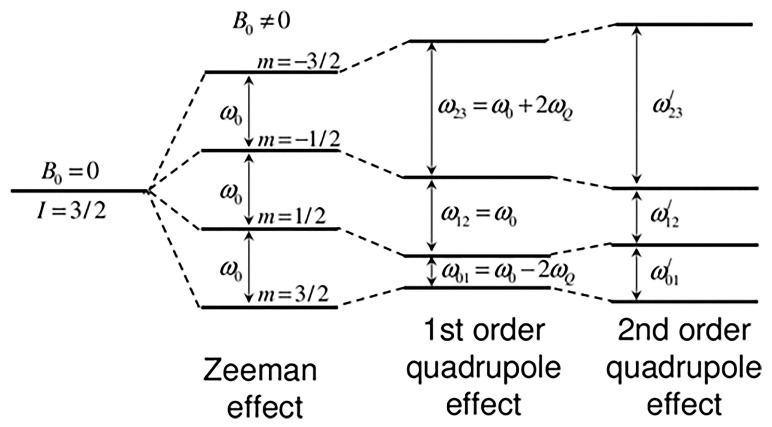
Schematic diagram of the energy levels of a spin I = 3/2 nucleus in an external magnetic field B_0_ due to Zeeman interactions with additional shifts originating from the first- and second-order quadrupole interactions. Reprinted with permission from [32]. Copyright 2015, The American Physical Society, Washington, DC, USA.

^33^S NMR can provide important information about molecular structure and dynamics in the solid state; however, the extremely broad powder patterns ranging from hundreds of kHz to tens of MHz due to large quadrupolar anisotropic interaction and the very long longitudinal relaxation hinder the routine application of this nucleus [1,31]. Figure 11 shows the energy levels resulting from the Zeeman first- and second-order quadrupolar interactions for a nucleus with I = 3/2 [32]. The first-order quadrupolar interaction does not affect the central transition. The satellite transitions are shifted by ±2ω_Q_, the exact amount of which is orientation-dependent. The second-order quadrupolar interaction moves the energy levels by equal and opposite amounts and strongly affects the satellite transitions 1/2 ↔ 3/2 and −3/2 ↔ −1/2, thus resulting in extremely broad resonances. 

There are several methodologies that have been developed for the acquisition of ^33^S NMR spectra in the solid state:

(i) Static spectra: ultra-wideline Carr Purcell Meiboom Gill (CPMG) technique.
(ii) Magic-angle spinning observation of the central transition: effects of B_o_ and population transfer.
(iii) Dynamic nuclear polarization (DNP) in NMR.
(iv) Indirectly detected satellite transition via saturation of the proton reservoir.
(v) Zero-field and frequency/field-swept solid-state NMR.

A synopsis of (i) to (v) will be given below.

### 8.2. Static Spectra-Ultra-Wideline Carr Purcell Meiboom Gill (CPMG) Technique

**Figure 12:**
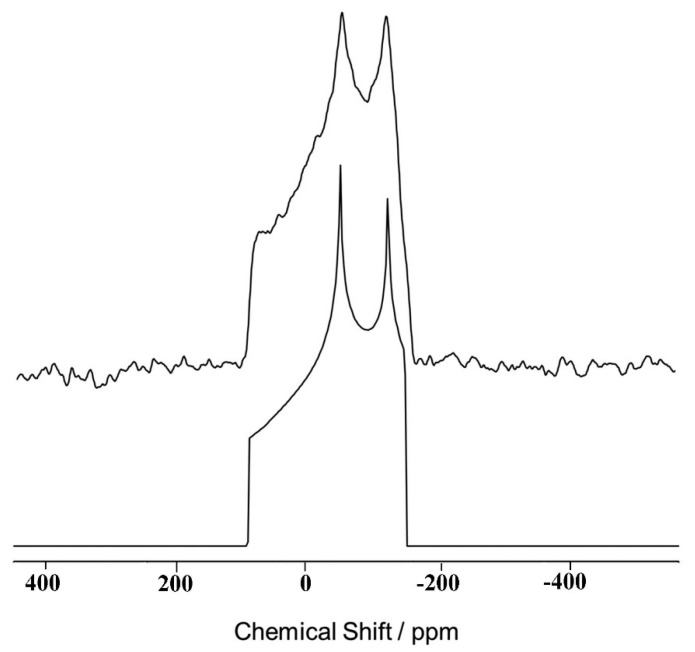
Static 61.42 MHz ^33^S NMR spectrum of polycrystalline [^33^S]-enriched taurine (10 mg) using ^1^H CW decoupling with Oldfield spin-echo technique, SW = 250 kHz, relaxation delay = 3 s, NS = 10,000. Adopted with permission from [9]. Copyright 2023, John Wiley & Sons Ltd., Chichester, West Sussex, UK. The chemical shift range has been corrected relative to saturated (NH_4_)_2_SO_4_ in D_2_O (0 ppm), according to IUPAC recommendation [5].

Experimental ^33^S static NMR spectra at 18.8 T of polycrystalline [^33^S]-enriched taurine were recorded, and the line shapes, which cover a range of ~15 kHz, were used to calculate the following parameters: δ_iso_ = −2(3) ppm, δ_11_ = 108(8) ppm, δ_22_ = −35(8) ppm, δ_33_ = −78(8) ppm, *C_Q_* = 1.39(6) MHz, and $n_{Q}$ = 0.65(4) (Figure 12) [9]. The small *C_Q_* value of the ${SO}_{3}^{-}$ group may be attributed to the high degree of symmetry even in the polycrystalline state.

For resonances broader than about 150 kHz, due to large *C_Q_* values, it is difficult to accurately record lineshapes with a single pulse technique. To overcome the problem, several approaches have been proposed to carry out spin-echo experiments of broad band excitation and refocusing at several frequencies to map out the lineshape [33]. These approaches are time-consuming; nevertheless, Halat et al. [34] performed successful ultra-wideline quadrupolar Carr-Purcell-Meiboom-Gill (QCPMG) ^33^S NMR experiments at natural abundance to investigate $S^{2^{-}}$ sulfide and $S_{2}^{2^{-}}$ disulfide units of the Li-ion battery conversion of the NbS_3_ electrode. Seventeen variable offset cumulative spectra were acquired at 20 T with a step size of 1970 ppm (128.5 KHz) in order to cover a spectral width of ~2.6 MHz. Spectral features corresponding to both $S^{2^{-}}$ and $S_{2}^{2^{-}}$ with significantly different *C_Q_* values were identified and supported with DFT calculations (see Section 9 on Computations).

### 8.3. Magic Angle Spinning Observation of the Central Transition: Effects of B_o_ and Population Transfer

The 1/2 ↔ −1/2 central transition of non-integer spins, such as ^33^S, is not affected by first-order broadening. Consequently, the central transition appears as a relatively sharp peak at the center of the satellite transitions, which are often too broad to be detected. In several cases, however, the second-order effect of ^33^S quadrupolar interactions contributes significantly to the line broadening of the central transition. MAS improves resolution but does not sufficiently remove anisotropic broadening. Thus, each distinct asymmetric ^33^S electronic environment will give rise to a second-order quadrupolar power pattern under MAS, which can be several KHz wide, thus resulting in significant signal overlapping. The two main developments that have made ^33^S solid-state NMR feasible are the availability of ultra-high magnetic field instruments and methods to enhance sensitivity via population transfer [35]. 

Second-order quadrupolar broadening, A, is inversely proportional to the strength of the static B_o_ field:

(9) $$A=\frac{3}{64}\frac{C_{Q}^{2}}{\frac{\gamma }{2\pi }{Β}_{ο}}$$

**Figure 13:**
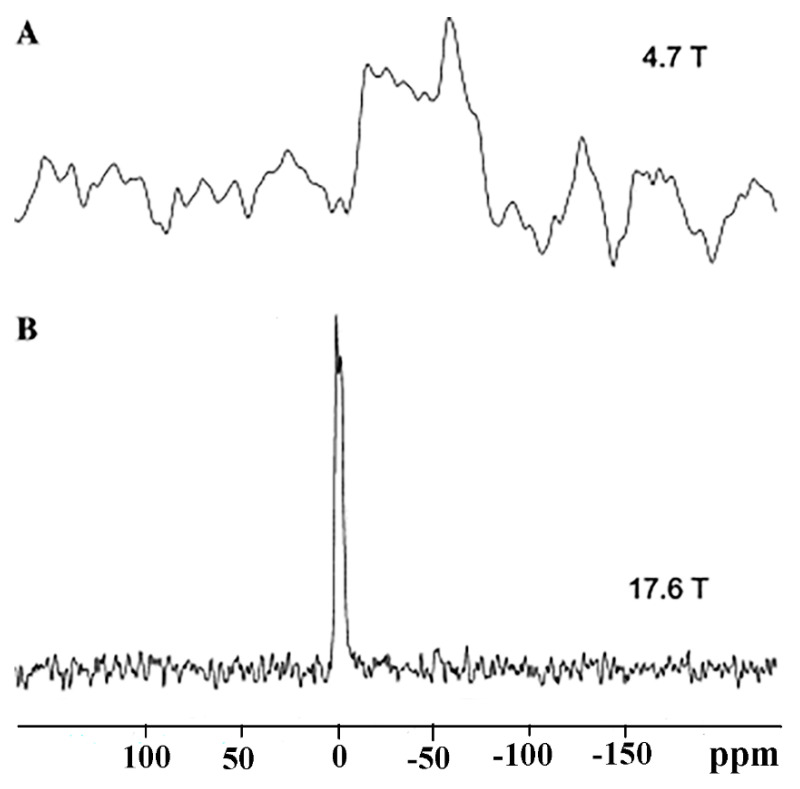
Solid-state ^33^S MAS NMR spectra of KAl(SO_4_)_2_·12H_2_O: (**A**) acquired at 4.7 T in 2 days using a 7 mm rotor with 215 μL sample volume; (**B**) acquired at 17.6 T in 1 h using a 5 mm rotor with 90 μL sample volume. Adopted with permission from [36]. Copyright 2004, Elsevier, Inc., Amsterdam, Netherlands. The chemical shift range has been corrected relative to saturated (NH_4_)_2_SO_4_ in D_2_O (0 ppm), according to IUPAC recommendation [5].

The ^33^S linewidth, therefore, is expected to be significantly reduced with a concomitant enhancement in sensitivity. Figure 13 shows the significant advantages of recording the spectrum of KAl (SO_4_)_2_·12H_2_O at 17.6 T relative to that of 4.7 T [36]. 

**Figure 14:**
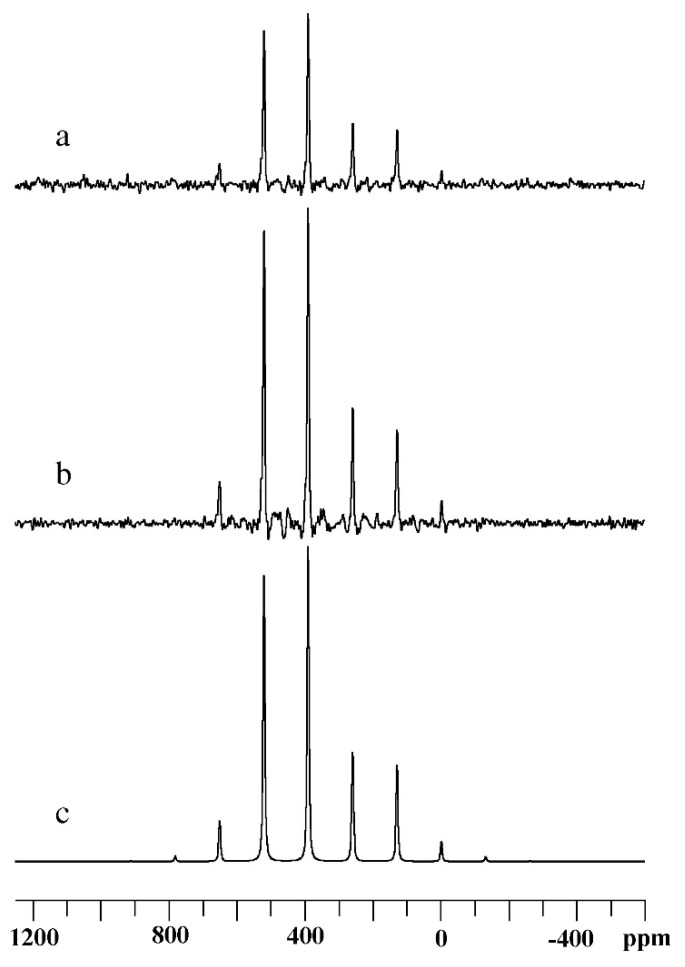
Natural abundance ^33^S MAS NMR spectra (*ν*_r_ = 6 kHz) of (NH_4_)_2_MoS_4_ displayed on an absolute intensity scale for (**a**) without polarization transfer and (**b**) with hyperbolic secant (HS) polarization transfer. Both spectra were observed using single-pulse excitation and identical experimental conditions, i.e., 48,000 scans, 1 s relaxation delay, and 13.3 h. (**c**) Simulation based on the NMR spectrum in (**b**). Adopted with permission from [37]. Copyright 2008, Elsevier, Inc., Amsterdam, Netherlands. The chemical shift range has been corrected relative to saturated (NH_4_)_2_SO_4_ in D_2_O (0 ppm), according to IUPAC recommendation [5].

Hansen et al. [37] reported significant sensitivity enhancements of 1.7 to 2.3 with the use of pairs of inversion pulses to induce selective polarization transfer between the four ^33^S energy levels. The method was applied to surface ions and tetrathio metalates with quadrupolar nuclear parameter $C_{Q}$ values in the range of 0.3 to 1.2 MHz (Figure 14). The use of the WURST (wideband uniform rate smooth transaction) technique does not require a priori knowledge of the $C_{Q}$ and $n_{Q}$ values. 

### 8.4. Dynamic Nuclear Polarization (DNP) NMR

**Figure 15:**
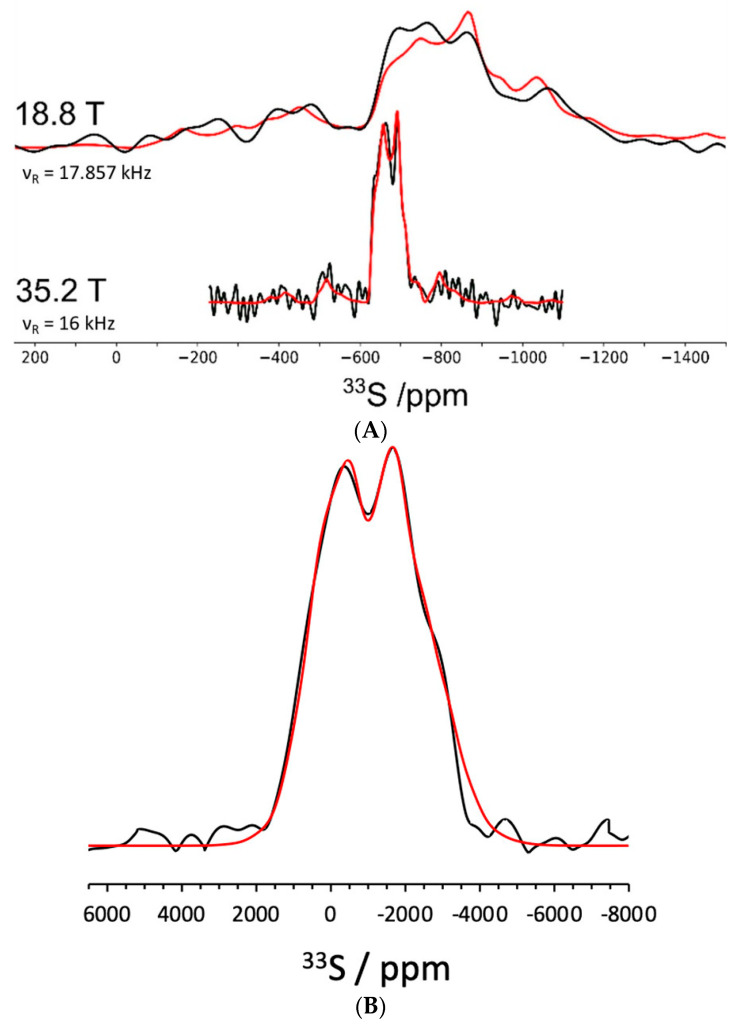
(**A**) Experimental (black) and simulated (red) conventional ^33^S direct excitation NMR spectra of ZnS·DDA NPls acquired at 18.8 T (top) and 35.2 T (bottom). The spectra are the FT of the sum of QCMPG echoes. (**B**) Experimental (black) and simulated (red) DNP-enhanced ^1^H → ^33^S D-RINEPT variable offset cumulative spectrum of ZnS·DDA NPls impregnated with 13 mM TEKPol solution in TCE acquired at 9.4 T and 105 K with ν_R_ = 13.89 kHz. The spectrum is the sum of four sub-spectra obtained by the FT of the sum of QCMPG echoes. Reprinted with permission from [39]. Copyright 2023, The American Chemical Society, Washington, DC, USA.

DNP has been extensively utilized to increase the sensitivity of NMR by several orders of magnitude and involves the transfer of polarization from highly polarized electron spins to nearby nuclear spins in the sample [38]. Microwaves at a specific frequency cause transitions between coupled electron-nuclear spin states, resulting in nuclear spin polarization. Bellan et al. [39] performed the first ^33^S DNP-NMR of ZnS nanoplatelets in order to characterize sulfur vacancies at the atomic scale. Figure 15A shows ^33^S direct detection NMR spectra at 18.8 and 35.2 T with a total experimental time of 6 days 8 h and 6 h 20 min, respectively. Simulation of a single central transition resulted in δ_iso_ = −621 ppm, $C_{Q}$ = 5.2 MHz, and $n_{Q}$ = 0.40. On the contrary, the ^67^Zn NMR showed the presence of three distinct central transitions due to the larger chemical shift range and, thus, sensitivity to the electronic environment. Application of the DNP method using a dipolar-mediated refocused INEPT technique (D-R INEPT) [40,41], in conjunction with QCPMG detection, resulted in a significant sensitivity enhancement by orders of magnitude of ^33^S NMR, with a total experimental time of 6 h for the four subspectra (Figure 15B). The DNP-NMR spectrum was acquired at 105 K using the compound TEKPol as a polarizing agent at 9.4 T.

### 8.5. Indirectly Detected Satellite Transition via Saturation of the Proton Reservoir

Satellite transitions result, in most cases, in extremely broad resonances that are typically invisible for the low-natural abundance nuclei, like ^33^S. Recently, Frydman and collaborators introduced a novel method for enhancing satellite transitions that is based on the progressive saturation of the proton reservoir (PROSPR) in static solids [42]. The PROSPR method includes a looped cross-polarization/spin diffusion process that progressively depletes the abundant ^1^H polarization in solid samples. This ^1^H depletion indirectly maps the NMR spectra of the heteronuclei as an attenuation of the abundant ^1^H NMR signals. The achievable sensitivity enhancement with conventional NMR instruments and modest hardware modifications at room temperature is orders of magnitude greater in comparison to directly observed experiments [42]. 

**Figure 16:**
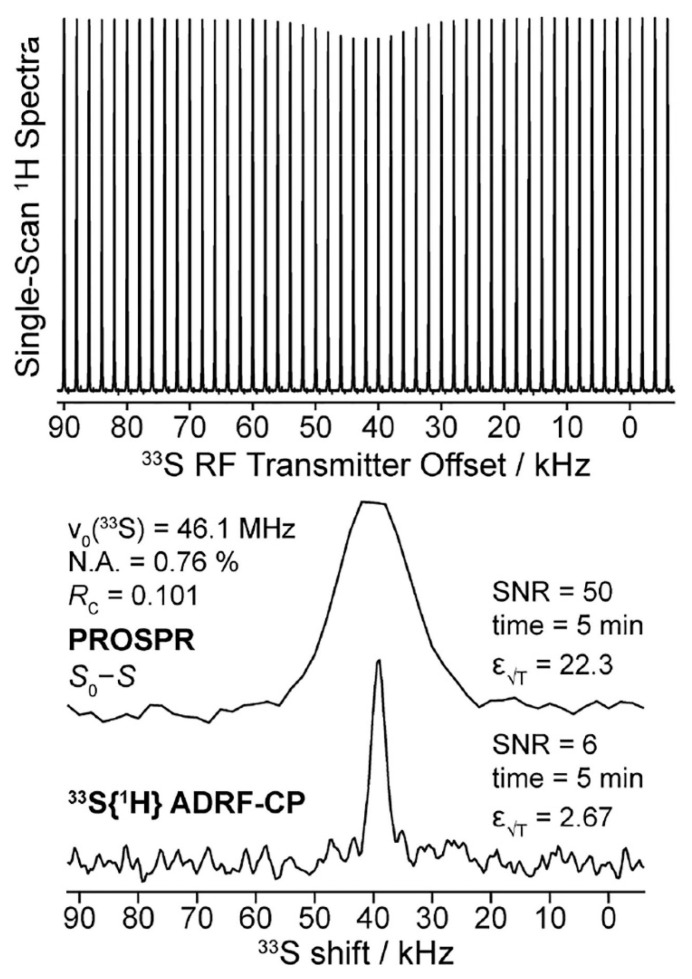
^33^S NMR spectra collected with the PROSPR and ADRF-CP pulse sequences. Reprinted with permission from [42]. Copyright 2021, The American Chemical Society, Washington, DC, USA.

Figure 16 illustrates the excellent sensitivity advantages of the PROSPR ^33^S NMR spectrum of the ammonium sulfate (NH_4_)_2_SO_4_) in natural abundance. The PROSPR NMR patterns appear with increased line width, which can be attributed to the limited spectral selectivity of the cross-polarizing ^33^S r.f. pulse. The use of an adiabatic demagnetization in the rotating frame (ADRF) cross-polarization scheme results in more effective multiple repeats and improved heteronuclei (^33^S) spectral selectivity (Figure 16).

### 8.6. Zero-Field and Frequency-Swept NMR in the Solid State 

**Figure 17:**
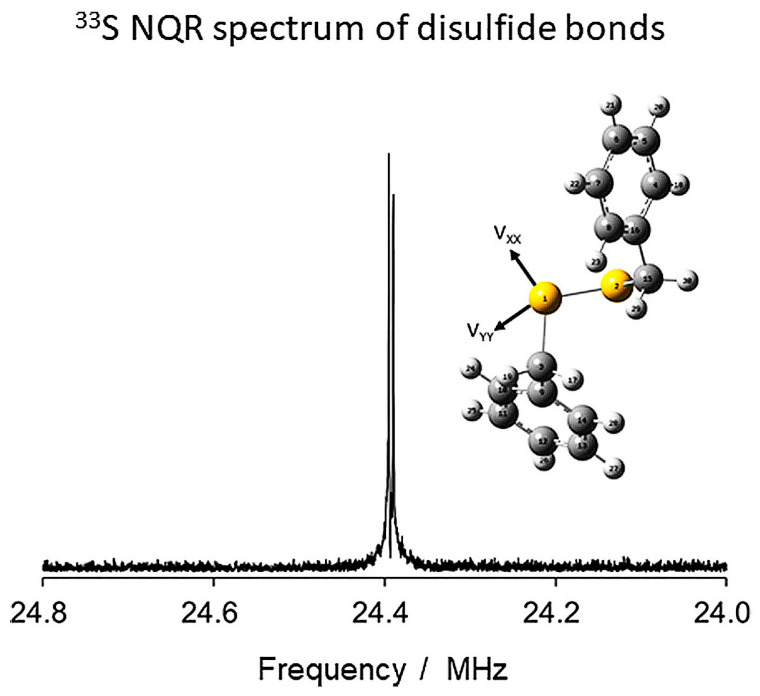
Sulfur-33 NQR spectrum of [^33^S]-dibenzyl disulfide, acquired at T = 200 K. Hydrogen, carbon, and sulfur in white, grey, and yellow, respectively. Reprinted with permission from [45]. Copyright 2019, Elsevier Inc., Amsterdam, the Netherlands.

In the case of organosulfur molecules or covalent bonds, as in the case of sulfur elements, the $C_{Q}$ values are reported to be more than 40 MHz. Thus, even with the highest currently available magnetic fields up to 28.8 T, the ^33^S NMR spectra would only be detected with frequency-swept or field-swept solid-state methods [43]. An alternative method, zero-field solid-state NMR or nuclear quadrupole resonance (NQR) spectroscopy, has been proposed, which utilizes the electric field gradient at the ^33^S nucleus instead of an external magnetic field [43,44]. Figure 17 shows an excellent zero-field ^33^S NMR spectrum of ^33^S-enriched dibenzyl disulfide, with $C_{Q}$ = 46.8 MHz and $n_{Q}$ = 0.48 [7], which was recorded with a total experimental time of only 1 min [45]. The method does not provide chemical shifts; however, the $C_{Q}$ and $n_{Q}$ parameters strongly depend on the local electric field gradient tensor on the sulfur nucleus and, thus, are very important structural parameters at the atomic level.

### 8.7. Selected Applications in Transition Metal Complexes, Ferroelectric, and Ferromagnetic Materials

Jakobsen et al. [46] utilized the WURST inversion pulses of the two ±3/2 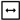 ±1/2 satellite transitions of the +1/2 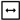 −1/2 central transition, which resulted in an increase in the S/N ratio by a factor ≥ 2 [37] to investigate disordered tetrathio transition metal anions. The use of two MAS frequencies (5.0 and 10.0 KHz) at 14.1 T in combination with static QCPMG at 19.6 T allowed the analysis of complex spectra of disordered Re$S_{4}^{-}$ anions at natural abundance. The nuclear quadrupolar parameters, $C_{Q}$ and $n_{Q}$, the chemical shift anisotropy parameters, δ_ani_ and η_ani_, and δ_iso_ of two different S sites of the four sulfur atoms in the Re$S_{4}^{-}$ anion were determined (Table 4). It was concluded that the use of high MAS speed resulted in narrow second-order line shapes for the central transition and, thus, improved precision of the CSA parameters.

^33^S MAS NMR (at 18.8 T), XANES, and Raman spectroscopy were applied to investigate sulfur speciation in iron-free and iron-poor glasses and, more specifically, to quantify the relative concentrations of S^2−^, S^6+^, and any intermediate oxidation states present in silicate glasses quenched from melts [47]. ^33^S NMR can be used to detect S^2−^ and S^6+^ in isotopically enriched samples with concentrations down to ~300 μg/g and long acquisition times of 1–2 days. However, both species have not been recorded simultaneously, although XANES and Raman showed the coexistence of S^2−^ and S^6+^ in the glass samples. It was concluded that ^33^S NMR can be applied to very specific synthetic S-rich systems but not to Fe-bearing natural compositions. 

**Figure 18:**
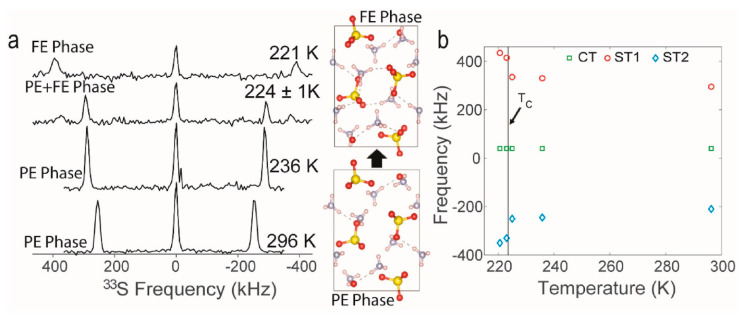
(**a**) Variable-temperature single-crystal ^33^S PROSPR measurements of the paraelectric to ferroelectric phase transition of ammonium sulfate; significant changes in the ST are lost in the CT spectra. (**b**) Temperature-dependence is shown by the ST and CT ^33^S resonance frequencies in these experiments. The structures shown in between (**a**) and (**b**) depict the changes presumed for the ammonium and sulfate (red/yellow balls) groups of (NH_4_)_2_SO_4_, upon going from the paraelectric phase (PE, lower inset) to the ferroelectic phase (FE, upper inset). Reprinted with permission from [48]. Copyright 2023, Elsevier, Inc., Amsterdam, Netherlands.

Frydman and collaborators [48] used the ^33^S PROSPR method to investigate ammonium sulfate, which undergoes a phase transition from its paraelectric (PE) to ferroelectric (FE) phase at T_c_ = 223.5 K. In the temperature range of 296–224 K, there is a monotonic increase in the *C_Q_* values from 0.58 MHz to 0.67 MHz. At T_c_ = 223.5 K, the spectrum showed the coexistence of both the PE and FE phases (Figure 18). The satellite transitions (STs) are much more sensitive to temperature changes of $C_{Q}$ than central transitions (CTs) and, thus, are of great importance in investigating dynamic processes over a wide range of time scales.

^33^S NMR measurements using ^33^S-entiched ferromagnetic EuS were performed in the range of 1.3 to 4.2 K [49]. The extrapolated NMR signal at T = 0 has a value of 12.73 MHz (≈39 kG). EuS displays T^1.71^ behavior above 4.2 K and T^2^ dependence below 4.2 K.

## 9. Computations of ^33^S NMR Parameters

### 9.1. Computational ^33^S NMR in the Gas and Liquid Phase 

Theoretical calculations of ^33^S NMR parameters, including the computation of ^33^S isotropic nuclear shieldings, spin-spin coupling constants involving the ^33^S isotope, and ^33^S nuclear quadrupole coupling constants, received reasonable attention; see review by Musio [3]. This was mainly for two reasons: calculations could help in identifying and elucidating the structural properties of the sulfur-containing molecules and, on the other hand, provide important information on the electron distribution and bonding situation around the sulfur atom. However, quite a few papers deal with computational ^33^S NMR, mainly due to the lack of experimental data used to benchmark such calculations.

Generally, for the heavy elements, the available methods for the prediction of NMR parameters based on the Schrödinger equation become insufficient. In this case, the average orbital velocities of electrons in the vicinity of nuclei are close to the speed of light, giving rise to so-called relativistic effects such as spin-orbit coupling, also known as *zitterbewegung* (Darwin term), and mass-velocity correction, which can essentially affect NMR spectroscopic parameters. In general, relativistic effects on the NMR parameters become noticeable for the atoms beginning with the third period of the Periodic Table; however, for sulfur, such effects are expected (but not yet documented) to be fairly negligible. In one of the early papers, Kupka and coworkers [50] reported DFT calculations of ^33^S NMR chemical shifts supported by experimental Raman and NMR data for thiophene and 3-methylthiophene. The Raman spectra of liquid thiophene were re-examined, and the performance of a hybrid B3PW91 functional used in combination with Pople’s basis set 6-311++G(d,p) was benchmarked at the ab initio restricted Hartree-Fock method. 

**Figure 19:**
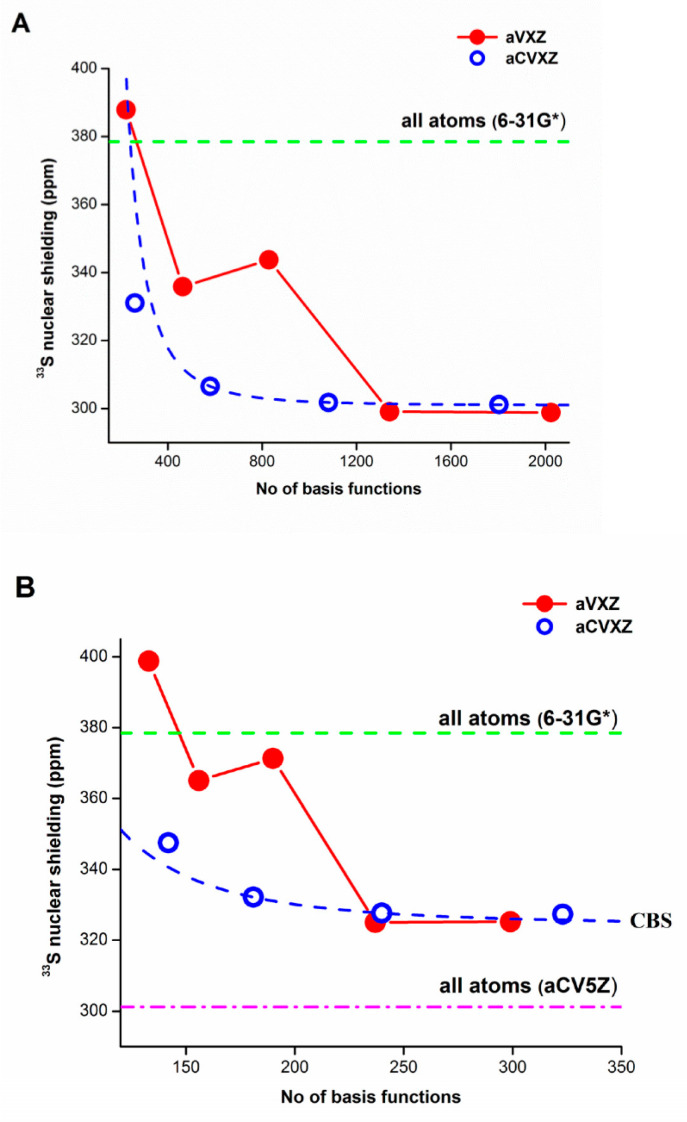
^33^S nuclear isotropic shielding constants of 2-thiouracil calculated at the (**A**)—B3LYP/aug-cc-pVXZ and B3LYP/aug-cc-pCVXZ levels of theory, and (**B**)—using the LDBS approach, where only the sulfur atom was described using either the aug-cc-pVXZ or aug-cc-pCVXZ while the 6-31G* basis set was applied on H, C, N, and O atoms [51].

In a much later paper by the same leading author [51], the ^33^S nuclear isotropic shielding constants of 2-thiouracil were calculated at the B3LYP/aug-ccpVXZ and B3LYP/aug-cc-pCVXZ levels of theory. Figure 19 compares the convergence patterns of the ^33^S isotropic shieldings for 2-thiouracil calculated at the B3LYP level with the aug-cc-pVXZ and aug-cc-pCVXZ basis sets. For the first family of basis sets, a considerable scatter of calculated values was observed, while a smooth convergence was found for the latter one. The same behavior was noticed when the Locally Dense Basis Set (LDBS) approach was employed. The change in estimated Complete Basis Set (CBS) values due to the LDBS approach was found to be about 10% of the CBS value when all atoms were described with the aug-cc-pVXZ or aug-cc-pCVXZ basis sets.

**Figure 20:**
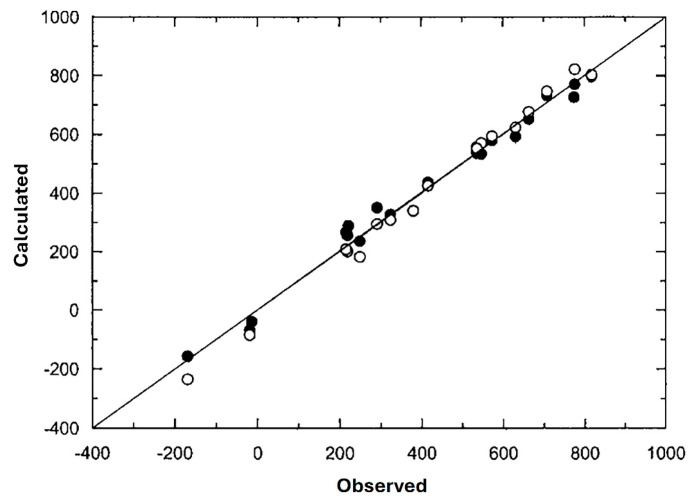
Calculated versus observed sulfur absolute shieldings (ppm) for the scaled DFT (solid circles) and EMPI (open circles) methods. Reproduced from Chesnut and Quin [53] with the permission of Wiley, Chichester, West Sussex, UK.

Bagno in his early paper [52] reported ^33^S NMR chemical shifts of a wide series of organic and inorganic sulfur-containing compounds, calculated at the DFT-GIAO level using the 6-311++G(2d,2p) basis set. Theoretical values were found to be in good agreement with the available experiment, with a few exceptions (mostly those for the charged species); see Table 5. Seven years later, Chesnut and Quin [53] reported a number of sulfur chemical shieldings (*σ*, ppm) evaluated at the scaled correlation level, including density functional theory, B3LYP/6-311+G(nd,p)//B3LYP/6-311+G(d,p), and modified MP2/6-311+G(nd,p) estimated infinite order Møller-Plesset theory with n = 2, the latter abbreviated as EMPI. The results of the ^33^S NMR chemical shieldings are exemplified in Table 6. Calculations spanned over the range of available sulfur shieldings showed an agreement with the experiment of about 3% of the whole shielding range (Figure 20). For the EMPI method, the authors used a particular mixture of RHF and MP2 approaches. In many cases, the Møller-Plesset series of corrections appeared to converge in a manner that allowed the infinite series to be summed, so that the EMPI shieldings could be represented by a particular combination of the RHF and MP2 contributions. The ^33^S NMR chemical shifts for fluoride, chloride, and bromide of the trimethylsulfonium ion and the *S*-methyltetrahydrothiophenium ion, in addition to the corresponding free cations, were also calculated within the same scaled DFT and EMPI approaches [54]. Experimental values were found to agree with the calculated values with the standard deviation of 35 ppm (3.5% of the shielding range) established in the previous paper [53] for a larger variety of sulfur compounds (Table 6).

Musio and Sciacovelli [55] calculated sulfur isotropic absolute shielding constants at the DFT level of theory (B3LYP/6-311++G(2d,p)) in the series of 2-substituted sodium ethanesulfonates, X–CH_2_–CH_2_–SO_3_Na (X = H, CH_3_, OH, SH, NH_2_, Cl, Br, NH_3_^+^). The diamagnetic contribution to the sulfur shielding constant was found to be constant, so that the observed deshielding of the ^33^S resonance induced by the electron-removing substituents could be primarily related to the variations of the paramagnetic contribution. It was also demonstrated that oxygen lone pairs and sulfur core 2*p* electrons could play an active role in determining the paramagnetic contribution to sulfur shielding. With regard to the linewidth variations, they could be ascribed primarily to changes in the nuclear quadrupole coupling constants. The same authors [56] calculated the ^33^S nuclear quadrupole coupling constants of 3- and 4-substituted benzenesulphonates and related charged species (both cations and anions) in the gas phase and in solution. The inclusion of the solvent effect in the calculations was found to be mandatory to reproduce experimental data. The solvent effect on the sulfur electric field gradient was shown to be due to electrostatic interactions. It was also shown that solvent interactions annihilated the Coulomb effect of the charge of the substituent and that those interactions caused a redistribution of electron density around the sulfur nucleus.

### 9.2. Computational ^33^S NMR in the Solid State

For computational ^33^S NMR in the solid-state, the available software and computational methods, the lineshape analysis and simulation of experimental data, together with the first principles calculations of NMR parameters, are of utmost importance. Some practical observations gained from a wide application of those approaches include, first of all, the choice of basis set and cluster approximation. Starting from a crystal structure, it is the accuracy of the unit cell parameters and atomic positions within the cell that determine the accuracy of calculated NMR parameters. The Gauge-Including Projector Augmented Wave (GIPAW) approach and its different modifications are particularly effective in reproducing NMR parameters. Although these approaches were initially aimed at periodic systems, there have been adaptations that allow parameters to be calculated when there is some atomic disorder in solid solutions and even in glasses. In particular, for glasses, Molecular Dynamics (MD) is effectively used to model the glass formation process, with the structural model further refined using DFT calculations, which then act as the input into GIPAW computation. For more details, see the recent review by Smith [31].

Wagler and coauthors [36] reported the solid-state ^33^S MAS NMR spectra of a variety of inorganic sulfates recorded at magnetic fields ranging from 4.7 to 18.8 T and magic angle spinning (MAS) at the natural abundance of the ^33^S isotope. A number of factors were considered when analyzing spectral linewidths, including magnetic field inhomogeneity, dipolar coupling, chemical shift anisotropy, chemical shift dispersion, and quadrupolar coupling. DFT calculations of the electric field gradient tensors revealed that the most significant contribution to the asymmetric electric field gradient around the sulfur nucleus was provided by the closest atoms, so that a general correlation could be observed between calculated and experimentally determined nuclear electric quadrupolar coupling constants.

**Figure 21:**
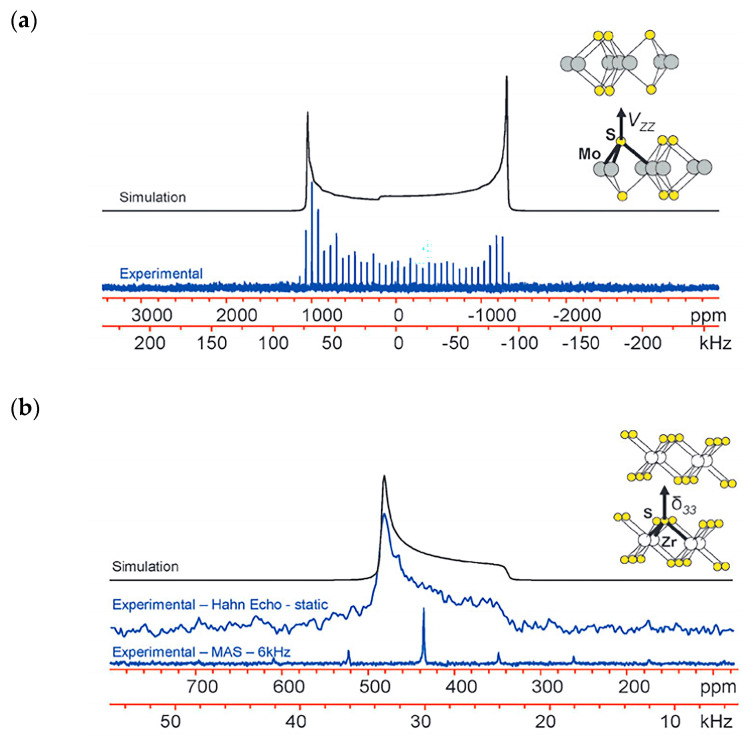
(**a**)—Experimental and simulated ^33^S static quadrupolar Carr-Purcell-Meiboom-Gill NMR spectra of 2H-MoS_2_ at 21.1 T; (**b**)—^33^S NMR spectra of 1T-ZrS_2_ at 21.1 T. Reproduced from Sutrisno et al. [57] with the permission of the Royal Society of Chemistry, London, UK.

Sutrisno et al. [57] demonstrated that a series of layered transition metal disulfides display a wide range of ^33^S quadrupole coupling constants and chemical shift anisotropy values. It was shown that the wide-line natural abundance solid-state NMR spectra of ^33^S in a less symmetric environment could readily be obtained at the ultrahigh magnetic field of 21.1 T and that, surprisingly, these closely related materials displayed a wide range of ^33^S quadrupole coupling constant and chemical shift anisotropy values, both experimental and calculated (Figure 21).

**Figure 22:**
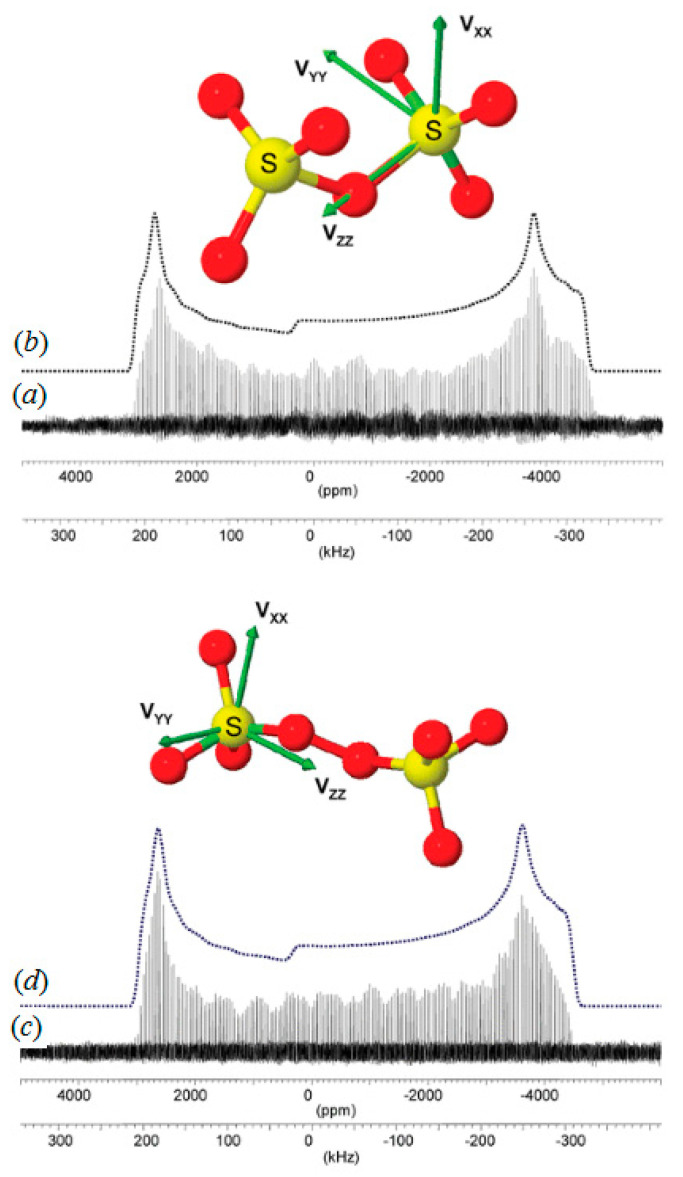
Top: ^33^S QCPMG spectra of K_2_S_2_O_7_ (top) and K_2_S_2_O_8_ (bottom) at 21.14 T. (**a**,**c**) Spectra have been obtained by coadding 14 equally offset subspectra. Each subspectrum is the result of 2000 accumulations with a 10 s delay. The upper traces (**b**,**d**) are the WSolids simulations of the spectra using a single site and a model of the central transition of spin 3/2. The inset demonstrates the calculated orientation of the EFG tensor. Sulfur and oxygen are shown in yellow and red, respectively. Reproduced from Moudrakovski et al. [58] with the permission of the American Chemical Society, Washington, DC, USA.

Moudrakovski et al. [58] performed the assignment of various sites and the relative orientations of the electron field gradient (EFG) tensors by means of quantum chemical calculations using the DFT method and Gauge Independent Atomic Orbitals (GIAO) on molecular clusters of about 100 to 120 atoms. CASTEP, a leading code for calculating the properties of materials from first principles, was used, which is specifically designed for periodic boundary conditions and a gauge-including projector-augmented wave pseudopotential approach. Although only semiquantitative agreement was observed between the experimental and calculated parameters, the calculations of ^33^S QCPMG spectra for K_2_S_2_O_7_ and K_2_S_2_O_8_ (Figure 22) appeared to be very useful in the interpretation of the experimental data. 

**Figure 23:**
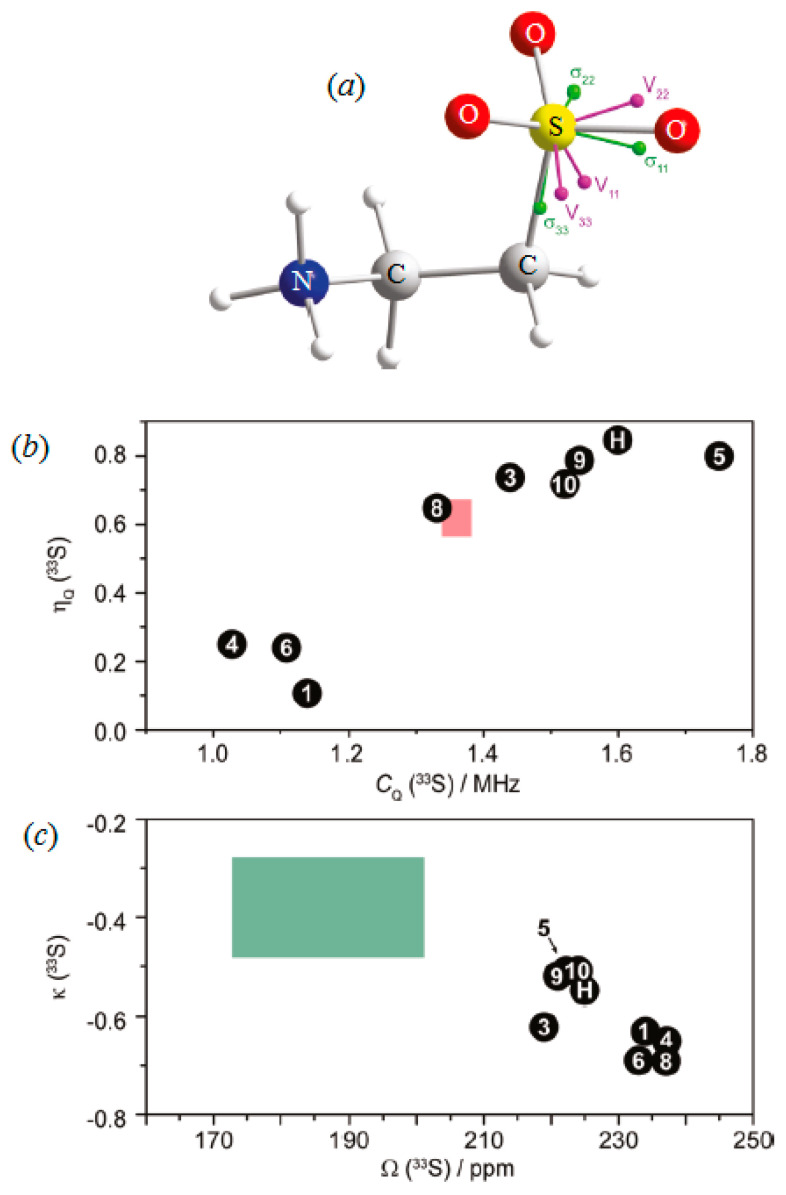
(**a**) Electric field gradient and chemical shift anisotropy tensor orientations in taurine; (**b**) the ^33^S quadrupolar interaction; (**c**) the ^33^S chemical shift anisotropy tensor orientations. The numbers n = 1–10 correspond to the CCSD code of the structure used (TAURINn), reproduced with minor editing privilege from O’Dell and Ratcliffe [59] with the permission of the American Chemical Society, Washington, DC, USA.

O’Dell and Ratcliffe [59] reported the crystal structure for taurine shown in Figure 23a. It was found that the most shielded component of the chemical shift anisotropy tensor, *σ*_33_, was aligned within 3° of the S-C bond, with *σ*_11_ pointing in the approximate direction of the S-O^2^ bond, while the largest component of the EFG tensor, *V*_33_, was aligned very close to the S-O^1^ bond (<5°), with *V*_22_ pointing in the approximate direction of the S-O^2^ bond. A combination of density functional and optimal control theory has been used by the authors to generate amplitude- and phase-modulated excitation pulses tailored specifically for the ^33^S nuclei, based on one of several reported crystal structures. This allowed the authors to perform accurate determinations of the ^33^S NMR interaction parameters at natural abundance and at a moderate magnetic field strength of 11.7 T. The ^33^S NMR parameters were then used to assess the accuracy of various proposed crystal structures specified in Figure 23b,c. Very recently, Masuda et al. [9] performed experimental (Figure 12) and computational studies of polycrystalline ^33^S-labeled taurine. The ^33^S chemical shift anisotropy parameters agree with those reported by O’Dell and Ractliffe [59]. It was concluded, however, that the orientations of the ^33^S electric field gradient and chemical shift tensors cannot be determined accurately since even small changes in the local asymmetry of the -${SO}_{3}^{-}$ group could introduce significant inconsistencies among the computational methods and molecular structures used. 

**Figure 24:**
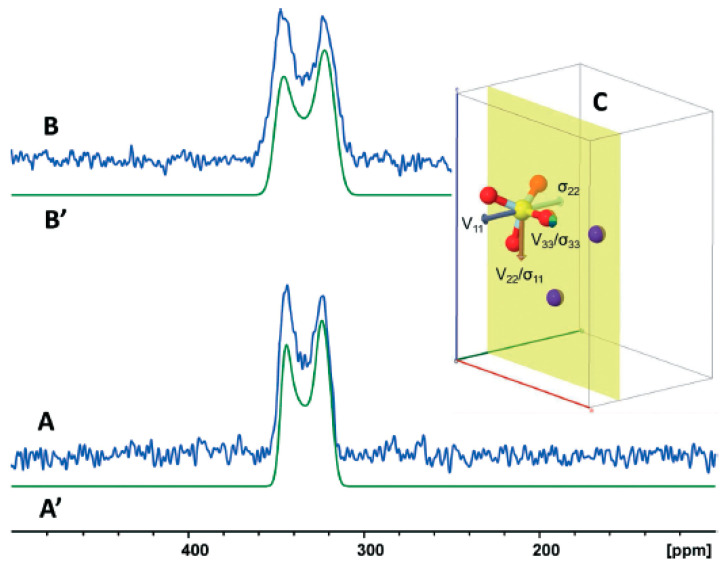
Experimental ^33^S stationary Hahn Echo spectra at 21.1 T (69.1 MHz) of Cs_2_SO_4_ (**A**) and Rb_2_SO_4_ (**B**). (**A**)—Experimental 5 kHz MAS Bloch Decay spectrum, (**A′**)—Simulation. (**A′**,**B′**) are corresponding simulation accounting for the EFG and CSA interactions. (**C**)—Representative portion of the Rb_2_SO_4_ unit cell showing the calculated orientations for the principal components of the EFG and CSA tensors. Sulfur, oxygen, and alkali metal are shown in yellow, red, and purple, respectively. The mirror plane present through sulfur is shown in yellow. Reproduced from Pallister et al. [60] with the permission of the Royal Society of Chemistry, London, UK. The chemical shift range reported relative to CS_2_ (0 ppm) has not been corrected relative to saturated (NH_4_)_2_SO_4_ in D_2_O (0 ppm), which is recommended by IUPAC [5].

**Figure 25:**
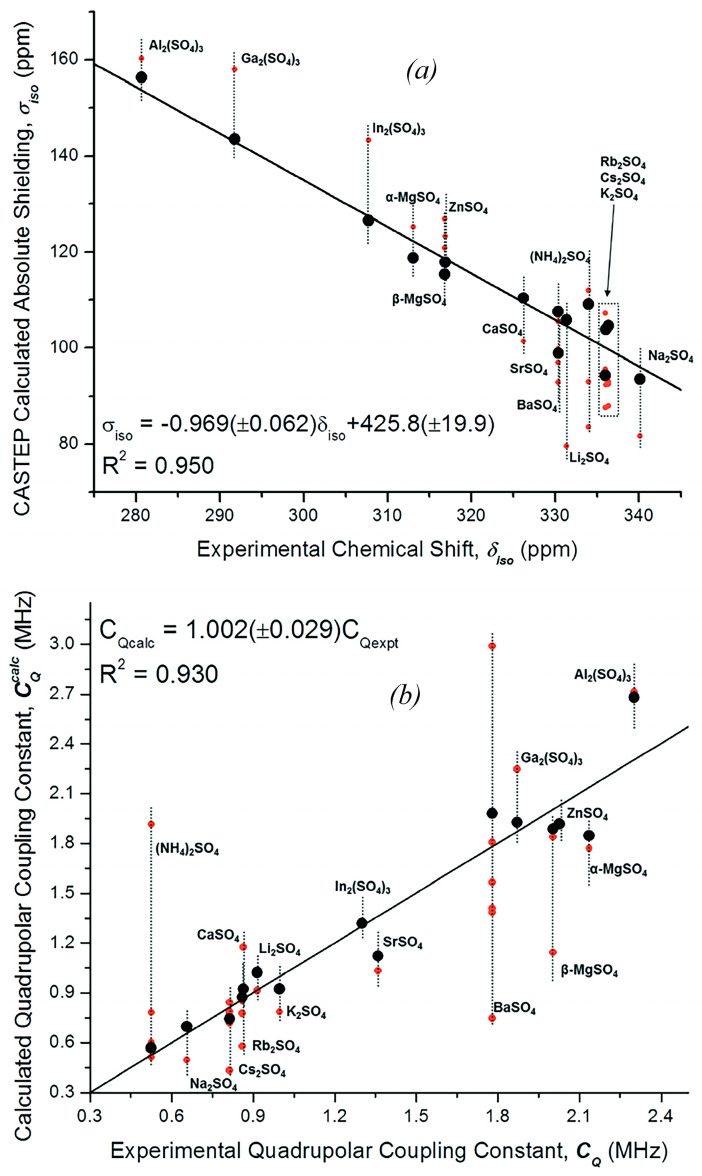
(**a**) Correlation between ^33^S experimentally determined chemical shift and calculated isotropic shielding constants for all known crystal structures of anhydrous sulfates. (**b**) Correlation between ^33^S experimentally determined quadrupolar coupling constant, *C_Q_*, and the calculated quadrupolar coupling constant, *C_Q_*^calc^ for the sulfate compounds. The red circles represent calculations for all structures, including those after the geometry optimization. The solid black circles indicate the structures that provided the best agreement with the calculated EFG. Reproduced with minor editing privilege from Pallister et al. [60] with the permission of the Royal Society of Chemistry, London, UK. The chemical shifts reported relative to CS_2_ (0 ppm) have not been corrected relative to saturated (NH_4_)_2_SO_4_ in D_2_O (0 ppm), which is recommended by IUPAC [5].

Pallister and coauthors [60] applied a combination of solid-state NMR, first principles calculations, and single crystal XRD to relate solid-state ^33^S NMR parameters obtained from a series of anhydrous sulfates of the elements of groups I–III. It was shown that the experimental ^33^S NMR spectra, exemplified in Figure 24, for Cs_2_SO_4_, and Rb_2_SO_4,_ were dominated by the quadrupolar interactions. Magnetic shielding constants and quadrupolar parameters for sulfur atoms were calculated using the plane wave pseudo-potential DFT. All calculated NMR parameters were found to be in very good agreement with the experimental results, which helped in the assignment of the stationary spectra. Indeed, reported correlations between ^33^S experimentally determined chemical shifts and calculated isotropic shielding constants and quadrupolar coupling constants were characterized by correlation coefficients R^2^ of 0.93–0.95 (Figure 25). Such a combined computational-experimental solid-state ^33^S NMR approach could aid in the assessment and interpretation of the unique crystallographic data.

**Figure 26:**
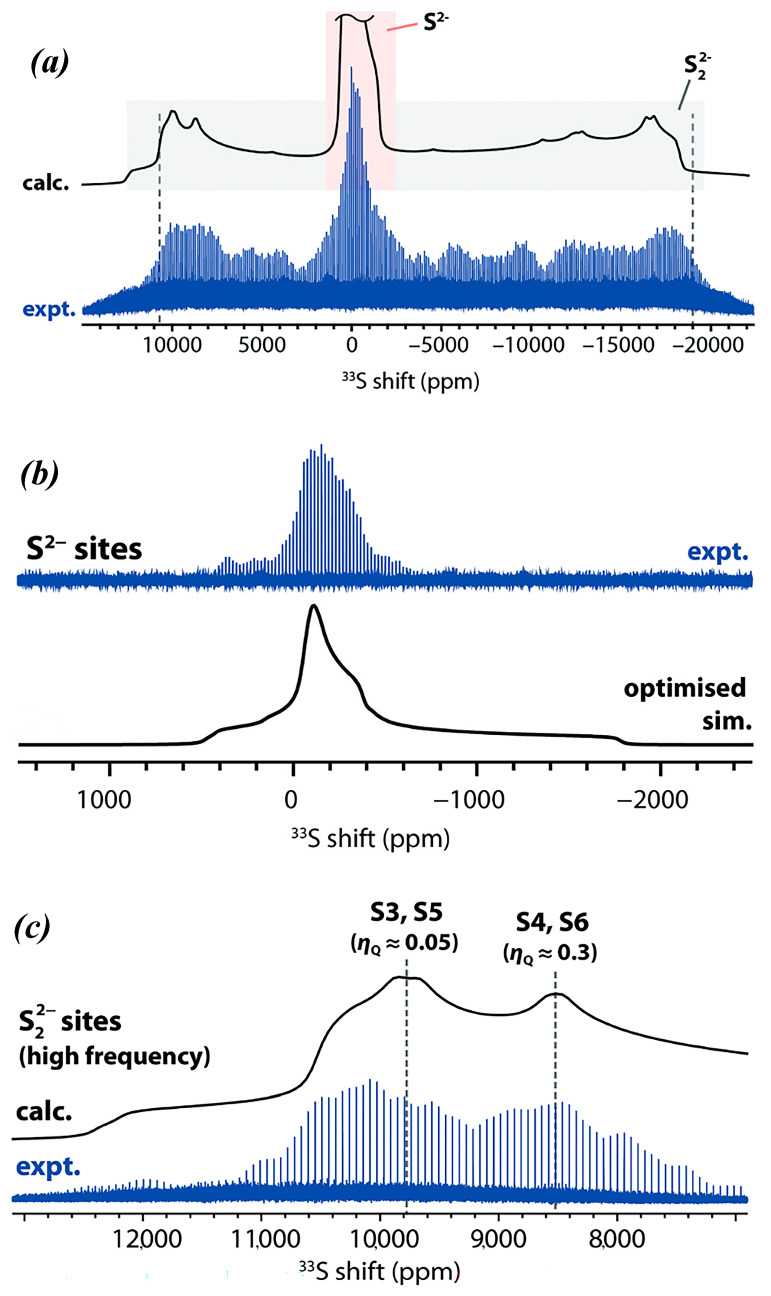
(**a**) Calculated ^33^S NMR spectrum at 20 T from the DFT parameters and experimental ultra-wideline static ^33^S spectrum of NbS_3_ acquired at 20 T using a frequency-stepping approach. Coloured boxes indicate the positions of the second-order quadrupolar broadened S^2−^ (pink) and S_2_^2−^ (grey) features; dotted lines show the discontinuities in the experimental spectrum; (**b**) Experimental static ^33^S NMR spectrum of NbS_3_ acquired at 20 T, centered at 0 ppm to acquire resonances arising from the S^2−^ environments, and simulated ^33^S spectrum using fitted parameters of the S^2–^ sites; (**c**) High-frequency region of the discontinuity arising from the S_2_^2−^ sites: the simulated ^33^S NMR spectrum using the DFT-calculated parameters and experimental static spectrum of NbS_3_ acquired at 20 T. Reproduced with minor editing privilege from Halat et al. [34] with the permission of the Royal Society of Chemistry, London, UK.

Halat and coauthors [34] reported ultra-wideline, high-field natural abundance solid-state ^33^S NMR spectra of the Li-ion battery conversion electrode NbS_3_, emphasizing the fact that it was the first ^33^S NMR study of a compound containing disulfide units. The large quadrupolar coupling parameters of about 31 MHz reported were consistent with the values obtained from the performed DFT calculations. As an illustration, several experimental and calculated at the DFT level ^33^S NMR spectra of NbS_3_ are presented in Figure 26.

**Figure 27:**
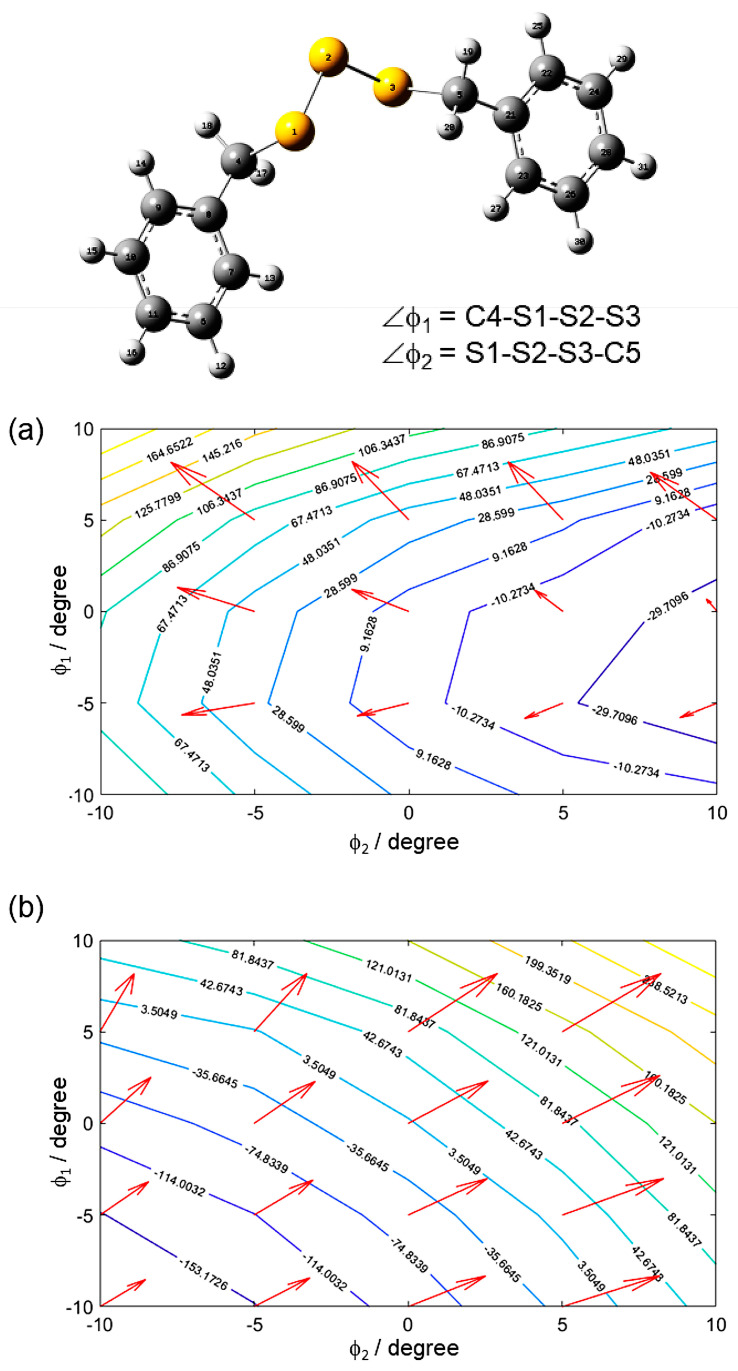
Contour plots of calculated *ν*_Q_ versus the two dihedral angles, *ϕ*_1_ and *ϕ*_2_, for (**a**) S^1^ and (**b**) S^2^ in dibenzyl trisulfide. The results for *ν*_Q_ were given as differences from those of *ϕ*_1_ = *ϕ*_2_ = 0° in kHz, and gradient values are expressed by red arrows. Hydrogen, carbon, and sulfur are shown in white, grey, and yellow, respectively. Reproduced with minor editing privilege from Yamada et al. [45] with the permission of Elsevier, Amsterdam, Netherlands.

Yamada and coworkers [45] performed experimental and theoretical investigations of the sulfur-33 EFG tensor of disulfide and trisulfide sulfur-sulfur bonds in the corresponding dibenzyl compounds. The orientations of the ^33^S EFG tensor were obtained by quantum chemical calculations. It was found that the largest EFG tensor component, *V_ZZ_*, was approximately perpendicular to the molecular plane, while the smallest component, *V_XX_*, was approximately 41° off the C-S bond. Extensive quantum chemical calculations were systematically performed by the authors to investigate the dependences of the ^33^S EFG tensors on changes in torsion angles in disulfide and trisulfide bonds (the latter are shown in Figure 27), indicating that analysis of the *ν_Q_* and *C_Q_* values potentially makes it possible to assign the secondary structures of crosslinking in rubber.

**Figure 28:**
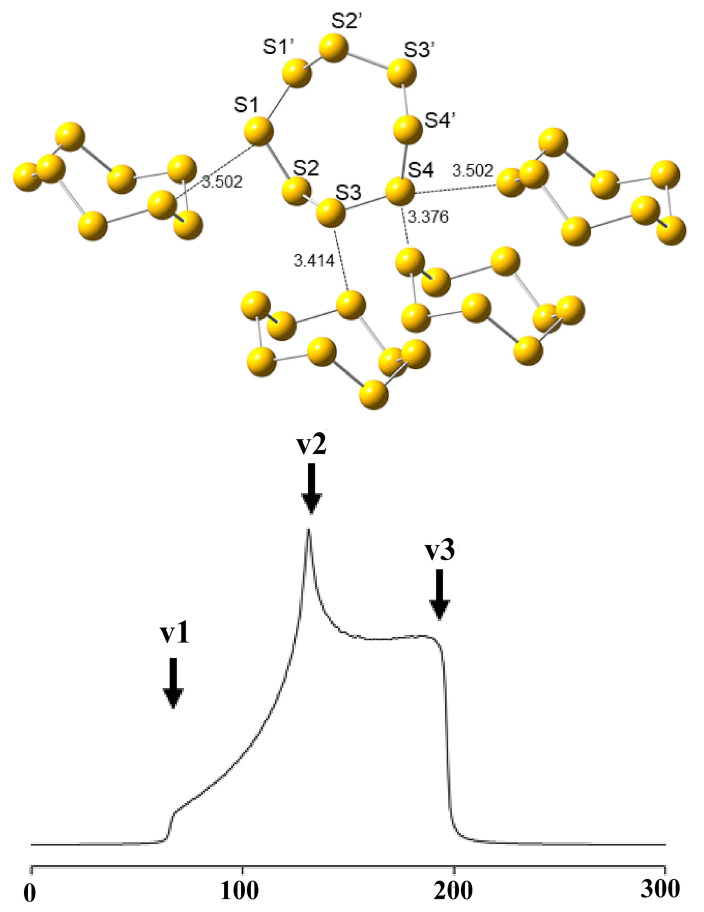
A simulated nutation echo NQR spectrum, which exhibits the three sharp singularities denoted as *ν*1, *ν*2, and *ν*3. The molecular structure of *α*-S_8_, determined by X-ray diffraction, is shown on top. Reproduced with minor editing privileges from Yamada [44] with the permission of Elsevier, Amsterdam, Netherlands.

Yamada [44] reported four ^33^S quadrupolar frequencies of the ^33^S-enriched elemental sulfur, *α*-S_8_, which were observed in the range of 23.122–23.280 MHz at 140 K and assigned based on the results of the quantum chemical calculations. The two-dimensional nutation echo ^33^S NQR techniques were carried out for each quadrupolar frequency, providing the ^33^S EFG tensor with the following information: the quadrupolar coupling constant and the asymmetry parameter. Quantum chemical calculations were also performed to complete the spectral assignment and to obtain the ^33^S EFG tensor orientations with respect to the molecular frame. As shown in Figure 28, the nutation NQR spectrum of *α*-S_8_ exhibits a powder pattern with three sharp singularities, denoted as *ν*1, *ν*2, and *ν*3 (*ν*1 < *ν*2 < *ν*3), and these frequencies can be used to obtain *ν_Q_* in a straightforward way.

Bellan and coauthors [39] reported ^33^S solid-state NMR experiments at ultra-high fields and DNP experimental data coupled with DFT calculations, which allowed, for the first time, to propose a structural model of ZnS nanoplatelets in full agreement with the observed optical and conductive properties. This study represented a step toward the use of NMR spectroscopy coupled with DFT calculations for a better understanding of the structure-property relationship of the metal sulfide nanomaterials.

The most recent paper by Kupka and coauthors [61] described the crystal and molecular structures of three new thiosulfonates. Theoretical analysis of molecular structure and vibrational IR, Raman, and NMR spectra was carried out. Theoretical structures closely resemble their geometry in the solid state. A profound influence of the substituent effect of amino and acetamido groups on the SO_2_S moiety in the *p*-position of the phenyl group was derived from theoretically computed quantitative aromaticity indexes based on geometric, magnetic, and electronic criteria. The substituent effect in the studied molecules, leading to chinoid structures of the phenyl ring and shortening of the C_Ar_-S bonds, was clearly pronounced both in crystal and DFT-calculated geometries. In addition, the presence of the oxygen-lone electron pairs on one side of the phenyl group was found to provide different aromaticities above and below the ring plane.

Combination of NMR studies at various magnetic fields, various signal enhancement techniques, line shape analysis/simulation of experimental data, and first principles calculations resulted in several isotropic, δ_iso_, and nuclear quadrupole coupling constants *C_Q_* and *n_Q_*_,_ which are compiled in Table 7. Chemical shifts of metal sulfides exhibited an extremely large chemical shift range of over 600 ppm (Figure 7), which was explained in terms of the crystalline ionicities and the effects of orbital overlap [1]. DFT calculations by Laskowski and Blaha [62] showed that the variation of δ(^33^S) in sulfides is mainly related to the presence of metal *d* states and their variation in the energy position within the conduction bands. The sulfate chemical shifts exhibit a narrow range of ~80 ppm, which is rather similar to that in solution, but with a wider range of *C_Q_* values of 0.53 to 2.3 MHz (Table 7). *C_Q_* values increase significantly to 10.6 MHz in KHSO_4_, 15.9 MHz in K_2_S_2_O_8,_ and 16.2 MHz in K_2_S_2_O_7_.

## 10. Conclusions and Prospects for Future Research

From the present review, it becomes evident that the number of reports of ^33^S NMR in solution and, to a lesser extent, in the solid state is extremely sparse. Nevertheless, several technical and methodological advances and the availability of elemental ^33^S (99.8 atom %) as the starting material for isotope enrichment are expected to give a significant impetus to ^33^S NMR, which are summarized below.

(1) Ultra-high-field instrumentation and high-sensitivity detection schemes will significantly enhance sensitivity and resolution in the case of: (i) small molecular-weight biological molecules with sulfur in a highly symmetric environment, which can be detected in enzymatic reaction products and in metabolomic studies [13]. (ii) Ligands, with *C_Q_* (^33^S) «10 MHz, bound to macromolecules and utilization, outside the extreme narrowing condition, of the m = 1/2 → m = −1/2 component, which is expected to result in significantly narrower resonances than the other component. Nevertheless, the prospect of recording meaningful NMR spectra for sulfur amino acid residues in proteins is rather poor. The use of ^77^Se as a substitution for sulfur for ^1^H detection in ^77^Se NMR is, probably, the method of choice [30]. (iii) Solid state since the second-order quadrupolar line width of the m = 1/2 → m = −1/2 central transition decreases linearly with the magnetic field [36]. In addition, significant sensitivity enhancement is expected with the use of selective polarization transfer between the four ^33^S energy levels [37].
(2) Spin-echo experiments of the broad band excitation of static solids and refocusing at several frequencies can be successfully used to map out line shapes resulting from sulfur sites with *C_Q_* (^33^S) > 10 MHz.
(3) Dynamic nuclear polarization (DNP) NMR would allow orders of magnitude sensitivity enhancement [39] and, thus, ^33^S NMR studies at natural abundance within a reasonable experimental time.
(4) The indirectly detected satellite transition via saturation of the proton reservoir (PROSPR) method and its variants [42,48] are expected to open new avenues of applications in investigating dynamic processes over a wide range of time scales.
(5) Zero-field NMR (NQR) and frequency/field-swept NMR. Although this method may sound rather esoteric for ultra-high-field NMR spectroscopists, it should be emphasized that successful spectra were recorded using zero-field NMR of selectively enriched ^33^S organosulfur compounds and models of cross-linked structures in rubber, with *C_Q_* values in the range of 42 to 46 MHz [43,44,45,63,64].
(6) The widespread availability of software and computational methods, the line-shape analysis and simulation of experimental data, and the great potentialities of ab initio calculations of NMR parameters will provide excellent means for structural and electronic information at the atomic level.

It should be emphasized, however, that for solid-state DNP-NMR experiments, specialized instrumentation is required, which is commercially available, or several components are necessary to upgrade more conventional wide-bore NMR instruments. Commercial zero-field NQR and frequency/field-swept NMR instruments are not widely spread and, in several cases, are available in specialized laboratories [45]. Fortunately, the highly promising indirectly detected satellite transition via saturation of the proton reservoir (PROSPR) method and its variants [42,48] can be applied with conventional NMR instrumentation with modest hardware modifications at room temperature, 

It is hoped that the present review will encourage a wide range of researchers to utilize ^33^S NMR spectroscopy as a powerful tool to provide structural, electronic, and dynamic information in a wide range of chemical, geological, and industrial applications. 

## Figures and Tables

**Table 1:** Magnetic properties and nuclear magnetic resonance parameters of the sulfur-33 nucleus [1,2,3,4,5].

Property		Units	Ref.
Spin number	3/2		
Nuclear magnetic moment	0.6432555 (10)	Nuclear magneton	[6]
	0.6432474 (107)		[7]
	0.643251 (16)		[8]
Magnetogyric ratio	2.055685	×10^−7^ Radians s^−1^ Tesla^−1^	
Resonance frequency			
(at 9.398 Tesla)	30.714	MHz	
Chemical shift range	ca. 1000	ppm	
Quadrupole moment	−0.0678	Electron m^2^	
Nuclear quadrupole coupling			
constant range	−10 to +42	MHz	
Relaxation times	<0.2	s	
Natural abundance	0.76	%	
Relative sensitivity per			
nucleus (^1^H = 1) ^a^	2.27 × 10^−3^		
Absolute relative sensitivity at natural abundance (^1^H = 1) ^b^	1.72 × 10^−5^		
Relative Receptivity to ^13^C	0.101		

^a^ Relative to proton, at constant field, for equal number of nuclei. ^b^ Product of relative sensitivity and natural abundance.

**Table 2:** Experimental ^33^S NMR chemical shifts and line widths of the representative sulfides, sulfoxides, and sulfones ^a^.

Cmpd.	Formula	Sulfides (X = S)		Sulfoxides (X = SO)		Sulfones (X = SO_2_)	
		Chemical Shift, ppm	Line Width, Hz	Chemical Shift, ppm	Line Width, Hz	Chemical Shift, ppm	Line Width, Hz
**1**	CH_3_–X–CH_3_	−428 (−9.5)	2500	−8 (325)	5500	−13 (320)	20
**2**	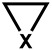	−573 (−240)	3220	−213 (120)	4950	−88 (245)	300
**3**	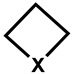	−302 (302)	4000	32 (365)	4950	−2 (331)	230
**4**	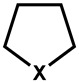	−336 (−3)	5500	27 (360)	2990	35 (368)	69
**5**	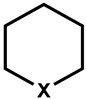	−363 (−30)	5500			−12 (321)	92

^a^ Chemical shifts are given in the SO_4_^2–^ and CS_2_ scales (the latter are given in parenthesis).

**Table 3:** Experimental ^33^S NMR chemical shifts and line widths of several representative sulfones.

Cmpd.	Formula	Solvent	^33^S NMR Chemical Shift, ppm		Line Width, Hz
			SO_4_^2−^ Scale	CS_2_ Scale	
	CH_3_–SO_2_–R, R =				
**6**	CH_2_Cl	CDCl_3_	−7.2	325.8	90
**7**	CHCl_2_	CDCl_3_	−0.2	332.8	280
**8**	CCl_3_	CDCl_3_	4.5	337.5	300
**9**	F	CHCl_3_	1	334	
**10**	Cl	CHCl_3_	2	335	1500
**11**	Br	CHCl_3_	−19	317	
**12**	I	CHCl_3_	−76	257	
	C_6_H_5_–SO_2_–R, R =				
**13**	Me	neat	−17	316	210
**14**	Et	neat	−9	324	460
**15**	*i*-Pr	neat	0	333	1100
**16**	*t*-Bu	neat	−3	330	1500
**17**	Me_2_SO_2_	DMSO-d_6_/CDCl_3_	−7	326	90
**18**	Et2SO_2_	DMSO-d_6_/CDCl_3_	10	343	200
**19**	*n*-Pr_2_SO_2_	DMSO-d_6_/CDCl_3_	7	340	130
**20**	*n*-Bu_2_SO_2_	DMSO-d_6_/CDCl_3_	7	340	320
**21**	*n*-Hex_2_SO_2_	DMSO-d_6_/CDCl_3_	9	342	400
**22**	(CH_2_=CH)_2_SO_2_	DMSO-d_6_/CDCl_3_	−26	307	60
**23**	(CH_2_=CH-CH_2_)_2_SO_2_	DMSO-d_6_/CDCl_3_	3	336	80
**24**	PhSO_2_Me	DMSO-d_6_/CDCl_3_	−20	313	120
**25**	PhSO_2_CH=CH_2_	DMSO-d_6_/CDCl_3_	−18	315	90
**26**	Ph_2_SO_2_	DMSO-d_6_/CDCl_3_	−23	310	130
**27**	(PhCH_2_)_2_ SO_2_	DMSO-d_6_/CDCl_3_	1	334	90
**28**	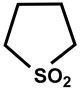	DMSO-d_6_/CDCl_3_	42	375	50
**29**	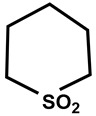	CS_2_	−12	321	92
**30**	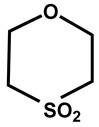	CHCl_3_	−18	315	100
**31**	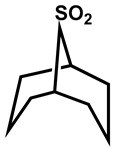	CS_2_	−8	325	190
**32**	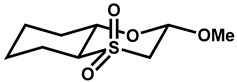	CHCl_3_	−10	323	200
**33**	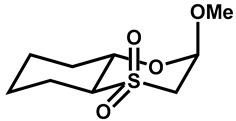	CHCl_3_	−20	313	200
**34**	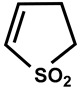	acetone-d_6_	39	372	8
**35**	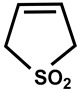	DMSO-d_6_/CDCl_3_	32	365	50
**36**	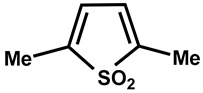	DMSO-d_6_/CDCl_3_	26	359	50
**37**	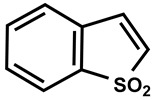	DMSO-d_6_/CDCl_3_	17	350	430
**38**	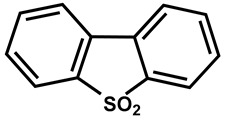	DMSO-d_6_/CDCl_3_	4	337	330

**Table 4:** ^33^S Quadrupole coupling ($C_{Q}$ and $n_{Q}$), chemical shift anisotropy parameters (δ_ani_ and η_ani_) and δ_iso_ for [(C_2_H_5_)_4_N][ReS_4_] determined from natural abundance WURST polarization transfer-enhanced ^33^S MAS and ^33^S static QCPMG NMR spectra. ^a^ Adopted with permission from [46]. Copyright 2010, Elsevier, Inc.

Entry Experiment	Sites	*C_Q_* (MHz)	$n_{Q}$	δ_ani_ (ppm)	η_ani_ (ppm)	δ_iso_ (ppm) ^b^
I						
14.1 T, MAS	S1	2.21	0.00	68	0.00	475 (808.0)
II						
14.1 T, MAS	S1	2.27	0.00	91	0.00	474.5 (807.5)
*ν*_r_ = 10.0 kHz	S2	2.48	0.18	67	0.37	433.4 (766.4)
III						
14.1 T, MAS	S1	2.21	0.00	83	0.00	476.8 (809.8)
*ν*_r_ = 5.0 kHz	S2	2.51	0.17	122	0.51	444.3 (777.3)
IV						
19.6 T, static	S1	2.21	0.00	91	0.00	474.1 (807.1)
spin–echo	S2	2.51	0.17	67	0.37	433.5 (766.5)

^a^ The optimum parameter set is shown in entry II. In the simulations for the S1 and S2 sites, the intensity ratio used was S1:S2 = 1:3, in accordance with the crystal structure for [(C_2_H_5_)_4_N][ReS_4_]. Due to the three-fold symmetry axis for S1, no error limits are given for $n_{Q}$. The error limits for the $C_{Q}$ values for both the S1 and S2 sites are within ±0.05 MHz. These error limits apply for all $C_{Q}$ and $n_{Q}$ values in Table 1, entries I–IV. ^b^ In parenthesis are the original chemical shifts reported relative to CS_2_ (0 ppm).

**Table 5:** ^33^S NMR chemical shifts (δ, ppm) of organic and inorganic sulfur compounds calculated at the DFT-GIAO/6-311++G(2d,2p) level as compared to the experiment. Compiled from Bagno [52].

Cmpd.	^33^S NMR Chemical Shift, ppm		Cmpd.	^33^S NMR Chemical Shift, ppm	
	Calc	Exp		Calc	Exp
OCS	−650	−594	SO_2_Cl_2_	−25	−46
Et_2_S_2_	−392	−501	Me_2_SO_2_	60	−12
thiophene	−168	−119	Me_2_SO_2_NMe_2_	−50	−9
H_2_S	−565	−503	MeSO_3_H	−40	−6
MeSH	−508	−458	MeSO_3_^−^	−40	−5
Me_2_S	−465	−428	MeSO_2_OMe	−35	2
Et–N=C=S	−640	−340	Na_2_S_2_O_3_	33	33
CS_2_	−386	−332	SOCl_2_	173	224
SF_6_	−253	−177	Ph–N=S=O	356	261
Me_2_SO	−100	−101	SO_2_	462	375

**Table 6:** The ^33^S NMR chemical shieldings (σ, ppm) of typical organic and inorganic sulfur compounds calculated at the DFT level (B3LYP/6-311+G(2d,p)//B3LYP/6-311+G(d,p), scaled by *k* = 0.871, and at the EMPI (RHF plus MP2/6-311+G(d,p)//MP2/6-311+G(d,p)) level, as compared to the experiment. Compiled from Chesnut and Quin [53].

Cmpd.	Method of Calculation		Exp
	Pure DFT	EMPI	
OCS	797.3	803.2	817
C_2_H_4_S	771.2	822.2	776
H_2_S	733.0	747.3	707.7
C_6_H_5_NCS	727.3		774
CH_3_SH	653.2	677.2	663.0
(CH_3_)_2_S	593.7	624.8	631
CH_3_SCN	580.2	595.0	573
CS_2_	558.4	552.4	536.1
C_6_H_5_SH	536.9		536
Tetrahydrothiophene	534.4	571.5	547
C_2_H_4_SO	437.6	426.3	416
C_2_H_4_SO_2_	351.7	295.8	291
SF_6_	339.9	340.9	379.9
Thiophene	328.5	309.6	324
C_6_H_5_SO_2_H	290.1		222
(CH_3_)_2_SO_2_	267.7	208.9	216
(CH_3_)_2_SO	257.0	202.0	219
Cl_2_SO_2_	236.7	183.0	249.4
C_2_H_5_SOCl	−39.1		−14
Cl_2_SO	−67.2	−84.6	−19
SO_2_	−157.1	−235.0	−169.7

**Table 7:** Isotropic ^33^S chemical shifts, δ_iso_, and nuclear quadrupole coupling constants $C_{Q}$ and $n_{Q}$ in the solid state (in brackets are the chemical shifts relative to CS_2_ at 0 ppm).

Compound	δ_iso_ ppm	*C_Q_* MHz	*n*_Q_
Li_2_S ^a^	−680		
Na_2_S ^a^	−671		
CdS (wurtzite) ^a^	−617		
ZnS (wurtzite) ^a^	−564		
MgS ^a^	−507		
SrS ^a^	−290.2		
BaS ^a^	−42		
Na_2_$SO_{4}$ ^a^	−3	0.82	
Cs_2_$SO_{4}$ ^a^	2	0.97	
Ca$SO_{4}$ ^a^	−7	1.0	
CaSO_4_·2H_2_O ^a^	−4	0.77	
Na_2_Ca$(SO_{4}{)}_{2}$ ^a^	5	0.71	
Na_2_Mg(SO_4_)_2_·4H_2_O ^a^	−12	2.2	
(NH_4_)Al(SO_4_)_2_·12H_2_O ^a^	0	0.53	
TlAl(SO_4_)_2_·12H_2_O ^a^	−1	0.56	
CsAl (SO_4_)_2_·12H_2_O ^a^	−2	0.53	
Li_2_${SO}_{4}$ ^b^	−2.1 (0.5) [330.9 (0.5)]	0.877 (0.050)	0.91 (0.05)
Na_2_${SO}_{4}$ ^b^	7.1 (1.0) [340.1 (1.0)]	0.655 (0.050)	0.0 (0.10)
K_2_${SO}_{4}$ ^b^	2.7 (0.5) [335.7 (0.5)]	0.959 (0.030)	0.42 (0.05)
Rb_2_${SO}_{4}$ ^b^	3.4 (0.6) [336.4 (0.6)]	0.860 (0.050)	0.42 (0.10)
Cs_2_${SO}_{4}$ ^b^	2.9 (1.0) [335.9 (1.0)]	0.813 (0.050)	0.4 (0.10)
(NH_4_)_2_${SO}_{4}$ ^b^	1.1 (0.4) [334.1 (0.4)]	0.520 (0.050)	0.85 (0.20)
a-Mg ${SO}_{4}$ ^b^	−19.9 (2.0 [313.1 (2.0)]	2.14 (0.05)	0.91 (0.05)
Ca${SO}_{4}$ ^b^	−6.8 (0.5) [326.2 (0.5)]	0.86 (0.05)	0.48 (0.10)
Sr${SO}_{4}$ ^b^	−2.6 (1.0) [330.4 (1.0)]	1.31 (0.05)	0.84 (0.10)
Ba${SO}_{4}$ ^b^	−2.6 (2.0) [330.4 (2.0)]	1.76 (0.05)	0.68 (0.05)
Al_2_(SO_4_${)}_{3}$ ^b^	−52.3 (2.0) [280.7 (2.0)]	2.32 (0.05)	0.55 (0.05)
(NH_4_)Al(SO_4_)_2_·12H_2_O ^b^	−2.6 [330.4]	0.106 (0.005)	0.05 (0.05)
KAl(SO_4_)_2_·12H_2_O ^b^	−1.2 [331.4]	0.633 (0.005)	0.20 (0.05)
KH${SO}_{4}$ ^b^	−3(<50) [330 (<50)]	10.6	0.37
K_2_S_2_$O_{7}$ ^b^	−13(<50) [320 (<50)]	16.2	0.1
K_2_S_2_$O_{8}$ ^b^	−3(<50) [330 (<50)]	15.9	0.1
[C_2_H_5_)_4_N] [ReS_4_] ^c^ S1	474.5 [807.5]	2.27 (0.05)	0.00
	433.4 [766.4]	2.48 (0.05)	0.18 (0.05)
ZnS (nanoplatelets) ^d^	−621	5.2	0.40
Nb$S_{3}$ ^e^ S1 S^2−^	−353 [−20]	4.4	0.38
S2	−576 [−243]	6.5	0.82
S3 $S_{2}^{2-}$	−289 [40]	30.3	0.04
S4	−220 [113]	30.5	0.27
S5	−275 [58]	30.1	0.05
S6	−221 [112]	30.3	0.29
[^33^S]-taurine ^f^	−2 (3)	1.39 (6)	0.65 (4)
α—$S_{8}$ ^g^		43.68	0.57
α—$S_{8}$ ^h^		44.6 (5)–45.7 (4)	0.30 (10)–0.49 (7)
[^33^S]-S-4 phenyl 4-toluene thiosulfonate ^i^		42.1	0.8
[^33^S]-dibenzyl disulfide ^j^		46.8 (6)	0.98 (7)

^a^ Complied from the review of Hinton [1]; ^b^ Complied from the review of Smith [31]; ^c^ Ref. [46]; ^d^ Ref. [39]; ^e^ Ref. [34]; ^f^ Ref. [9]; ^g^ Ref. [43]; ^h^ Ref. [44]; ^i^ Ref. [62]; ^j^ Ref. [45].

## Data Availability

Data are available from the authors.
